# Large-Scale Phenotyping of Livestock Welfare in Commercial Production Systems: A New Frontier in Animal Breeding

**DOI:** 10.3389/fgene.2020.00793

**Published:** 2020-07-31

**Authors:** Luiz F. Brito, Hinayah R. Oliveira, Betty R. McConn, Allan P. Schinckel, Aitor Arrazola, Jeremy N. Marchant-Forde, Jay S. Johnson

**Affiliations:** ^1^Department of Animal Sciences, Purdue University, West Lafayette, IN, United States; ^2^Department of Animal Biosciences, University of Guelph, Guelph, ON, Canada; ^3^Oak Ridge Institute for Science and Education, Oak Ridge, TN, United States; ^4^Department of Comparative Pathobiology, Purdue University, West Lafayette, IN, United States; ^5^USDA-ARS Livestock Behavior Research Unit, West Lafayette, IN, United States

**Keywords:** behavioral genomics, big data, digital agriculture, phenomics, genomic information, genomic selection, novel phenotypes, precision livestock

## Abstract

Genomic breeding programs have been paramount in improving the rates of genetic progress of productive efficiency traits in livestock. Such improvement has been accompanied by the intensification of production systems, use of a wider range of precision technologies in routine management practices, and high-throughput phenotyping. Simultaneously, a greater public awareness of animal welfare has influenced livestock producers to place more emphasis on welfare relative to production traits. Therefore, management practices and breeding technologies in livestock have been developed in recent years to enhance animal welfare. In particular, genomic selection can be used to improve livestock social behavior, resilience to disease and other stress factors, and ease habituation to production system changes. The main requirements for including novel behavioral and welfare traits in genomic breeding schemes are: (1) to identify traits that represent the biological mechanisms of the industry breeding goals; (2) the availability of individual phenotypic records measured on a large number of animals (ideally with genomic information); (3) the derived traits are heritable, biologically meaningful, repeatable, and (ideally) not highly correlated with other traits already included in the selection indexes; and (4) genomic information is available for a large number of individuals (or genetically close individuals) with phenotypic records. In this review, we (1) describe a potential route for development of novel welfare indicator traits (using ideal phenotypes) for both genetic and genomic selection schemes; (2) summarize key indicator variables of livestock behavior and welfare, including a detailed assessment of thermal stress in livestock; (3) describe the primary statistical and bioinformatic methods available for large-scale data analyses of animal welfare; and (4) identify major advancements, challenges, and opportunities to generate high-throughput and large-scale datasets to enable genetic and genomic selection for improved welfare in livestock. A wide variety of novel welfare indicator traits can be derived from information captured by modern technology such as sensors, automatic feeding systems, milking robots, activity monitors, video cameras, and indirect biomarkers at the cellular and physiological levels. The development of novel traits coupled with genomic selection schemes for improved welfare in livestock can be feasible and optimized based on recently developed (or developing) technologies. Efficient implementation of genetic and genomic selection for improved animal welfare also requires the integration of a multitude of scientific fields such as cell and molecular biology, neuroscience, immunology, stress physiology, computer science, engineering, quantitative genomics, and bioinformatics.

## Introduction

Animal welfare has increasingly relevant ethical, legal, and economic implications in livestock production around the world (Rushen et al., 2011; Koknaroglu and Akunal, 2013; Marchant-Forde, 2015; Grethe, 2017). Animal product consumers, and public in general, are becoming more interested in ensuring good welfare practices at all stages of the animal production chain, which has direct implications for the whole industry. In addition, poor welfare is associated with reduced animal productivity, longevity, poor meat quality, low reproductive performance, and high prevalence of diseases in herds or flocks (Cockram, 2002; Moberg, 2009; Miranda-de la Lama et al., 2013; Grethe, 2017; Croney et al., 2018a, b; Gonzalez-Rivas et al., 2020). This global importance of animal welfare is indicated by the inclusion of increasing numbers of species-specific and situation-specific animal welfare chapters in the OIE Terrestrial Animal Health Code (World Organization for Animal Health – OIE, 2019).

Historically, animal welfare has been defined under one of three over-arching, and intersecting themes or approaches (Fraser, 2008). These welfare approaches are biological functioning, natural behavior, and affective states. These three approaches overlap to provide a holistic overview of the welfare of the individual, and indicators of the three approaches should be taken into account in welfare assessments (Fraser et al., 1997). Nonetheless, defining measurable parameters that incorporate the underlying processes of all three approaches for multiple individuals under commercial conditions is challenging. This task is particularly difficult due to the context-dependent and conditional nature of the behavioral response and the affective state of the animals. However, the expression of natural behaviors is paramount to improve welfare due to species-specific behavioral needs (Duncan, 1998; Olsson et al., 2011). Specific behaviors (e.g., motivated behaviors) have an intrinsic value for animals, and the performance of these behaviors is necessary to achieve acceptable animal welfare (Duncan, 1998). Non-met behavioral needs and motivated behaviors results in frustration and can develop in distress and other emotional disorders (Mason, 2006; Keeling et al., 2011). Animals are sentience beings, and this implies that livestock can experience positive and negative affective states. For this reason, animal emotions are essential in welfare assessments, and improvements in animal welfare should promote positive affective states and reduce the negative ones (Broom, 2011; Mellor, 2016).

An often used approach in animal welfare assessment is based on the Five Freedoms (Brambell, 1965; McCulloch, 2013), which consists of the absence of negative welfare (thirst, hunger, and malnutrition; physical and thermal discomfort; pain, injury, and disease; fear; and distress) as well as the presence of positive welfare (e.g., freedom to engage in motivated behaviors; Broom, 1991; De Goede et al., 2013). These have been applied mostly in terms of housing and husbandry (Mellor, 2016). However, welfare assessments using the Five Freedoms examine on-farm environment by looking mostly at input or resource-based measures that usually describe the physical environment rather than at outcome or animal-based measures that directly refer to animal status (Butterworth et al., 2017). More recent focus has been on the development of animal-based indicators and expert opinion states that “*animal-based measures are the most appropriate indicators of animal welfare and a carefully selected combination of animal-based measures can be used to assess the welfare of a target population in a valid and robust way*” (European Food Safety Authority [EFSA], 2012).

Despite the fact that various countries have implemented regulations and legislation to ensure ethical animal treatment from birth to slaughter (Rushen et al., 2011), completely eliminating welfare issues (e.g., incidence of diseases, thermal, and metabolic stress) is still very challenging or impossible due to multiple factors, including: climate change, especially in outdoor systems (Cole et al., 2017); growing intensification of commercial production systems; group-housed animals in inadequate systems (negative interactions, e.g., due to aggressive behaviors, feather pecking, and cannibalism); antibiotic resistance (Mathew et al., 2007; Woolhouse et al., 2015); high disease prevalence (Zessin, 2006); and, to a lesser extent, genetic selection based on a limited number of production traits in some breeding programs or indirect genetic responses (Rauw et al., 1998, 2017). In this context, the implementation of selective breeding schemes to genetically modify the animals’ biological mechanisms and/or behaviors in ways that improve welfare in commercial systems is a promising route (Jensen et al., 2008; Turner, 2011; Croney et al., 2018b). This is likely to be achieved through selection and breeding of more resilient animals.

Genetic selection for improved welfare has been investigated and implemented in livestock species over the past few decades (Rodenburg and Turner, 2012; Canario et al., 2013). Several traits associated with animal welfare have been shown to be heritable (the majority of the estimates are in the range of 0.15 to 0.40; Tables 1–4), including: feather pecking (Buitenhuis et al., 2004; Muir et al., 2014; Grams et al., 2015), cannibalism (Rodenburg et al., 2008; Bennewitz et al., 2014), animal robustness (Muir et al., 2014; Rauw and Gomez-Raya, 2015; Friggens et al., 2017), overall mortality (Knol et al., 2002; Grandinson, 2005; Bolhuis et al., 2009), leg health (McLaren et al., 2016; Vargas et al., 2017), bone strength (Kapell et al., 2017; Oviedo-Rondón et al., 2017; Siegel et al., 2019), and immune response and disease resistance (Bishop and MacKenzie, 2003; Stear et al., 2012; Mallard et al., 2015; Schultz et al., 2020). Genetic and genomic selection for welfare traits, itself, is unlikely to solve all the welfare issues in commercial livestock operations. However, selective breeding is a complementary approach to other strategies (e.g., management, nutrition, housing, and biosecurity), which should result in permanent and cumulative gains in welfare (resilience) over generations.

**TABLE 1:** Heritability estimates for indicators of heat tolerance based on direct or indirect traits in pigs.

Indicator trait	Breed	Heritability	References
Feeding behavior	Crossbred animals (grow-finish)	0.02 to 0.21	Cross et al., 2018
Thermoregulation	Crossbred animals	0.39 to 0.83	Kim K. S. et al., 2018
Lactation performance	Large White (Lactating sows)	0.20 to 0.31	Gourdine et al., 2017
Thermoregulation	Large White (Lactating sows)	0.34 to 0.39	Gourdine et al., 2017
Body weight	Duroc (grow-finish)	0.23 to 0.26	Fragomeni et al., 2016
Hot Carcass weight	Crossbred animals (grow-finish)	0.17 to 0.18	Fragomeni et al., 2016
Farrowing rate	Large White and crossbred sows	0.02 to 0.08	Bloemhof et al., 2012
Carcass weight	Terminal crossbred (grow-finish)	0.14 to 0.51	Zumbach et al., 2008

**TABLE 2:** Heritability estimates for traits derived from Automated Milking Systems (AMS; milking robots) in Holstein (HO), Norwegian Red (NR), and Swedish Red (SR) cattle.

Trait	Measurement protocol or trait definition	Breed	Heritability (SE)	References
Attachment failures (%)	Proportion of milkings with at least one attachment failure	HO, SR	0.21 (0.07)–0.31 (0.07)	Carlström et al., 2016
Average flow rate (kg/min)	Average of the milk flows measured for each quarter within 1 milking	HO, SR	0.37 (0.06)–0.48 (0.08)	Carlström et al., 2013
Average milk flow	Average milk flow in kg/min	HO	0.25 (0.07)	Viviana Santos et al., 2018
Box time	Time from when a cow enters the AMS to when it exits the milking unit	NR	0.27 (0.03)	Wethal and Heringstad, 2019
Box time (min)	Difference between begin and end time	HO, SR	0.21 (0.05)–0.44 (0.07)	Carlström et al., 2013
Distance front-rear (mm)	Average distance between the front and rear teat ends	HO	0.56 (0.02)–0.61 (0.02)	Poppe et al., 2019
Electrical conductivity from all four quarters	Electrical conductivity was used as an indicator reflecting the udder health status	HO	0.53 (0.09)	Viviana Santos et al., 2018
Electrical conductivity	Electrical conductivity (EC) measured as maximum (ECmax) and mean (ECmean)	HO	0.23–0.35 (0.03)	Wethal et al., 2020
Elevated mastitis risk	Please see the formula in the reference	HO	0.09 (0.04)	Wethal et al., 2020
Flow rate	Average kg of milk/min of milking time	NR	0.48 (0.04)	Wethal and Heringstad, 2019
Front teat distance (mm)	Distance between left and right front teat ends	HO	0.53 (0.03)–0.60 (0.02)	Poppe et al., 2019
Handling time	Difference between box time and milking time and sums the time before the milk starts flowing and the time from when the last teat cup was removed to the time the cow leaves the system	NR	0.05 (0.01)	Wethal and Heringstad, 2019
Handling time (min)	Difference between box time and milking time	HO, SR	0.05 (0.02)–0.15 (0.03)	Carlström et al., 2016
Incomplete milking (%)	Proportion of incomplete milkings out of all milkings throughout the lactation	HO, SR	0.02 (0.03)–0.06 (0.05)	Carlström et al., 2016
Incomplete milkings	Number of daily milkings with a minimum of one teat registered as incompletely milked	NR	0.01 (0.01)	Wethal and Heringstad, 2019
Interval between two consecutive milkings	Time span between two consecutive milkings	HO	0.07 (0.03)	Viviana Santos et al., 2018
Kick-offs	Daily number of milkings with at least one teat cup kick-off	NR	0.06 (0.01)	Wethal and Heringstad, 2019
Log-transformed handling time	Log of handling time	NR	0.07 (0.02)	Wethal and Heringstad, 2019
Log-transformed online cell count	Udder health indicator	HO	0.09 (0.03)	Wethal et al., 2020
Milk yield at a quarter basis: front left	Milk yield measured by the AMS	HO	0.19 (0.06)	Viviana Santos et al., 2018
Milk yield at a quarter basis: front right	Milk yield measured by the AMS	HO	0.05 (0.06)	Viviana Santos et al., 2018
Milk yield at a quarter basis: rear left	Milk yield measured by the AMS	HO	0.11 (0.06)	Viviana Santos et al., 2018
Milk yield at a quarter basis: rear right	Milk yield measured by the AMS	HO	0.08 (0.05)	Viviana Santos et al., 2018
Milking efficiency	Ratio of milk yield (kg) and box time (min)	NR	0.22 (0.03)	Wethal and Heringstad, 2019
Milking frequency	Recorded by the AMS	HO	0.14 (0.01)	Nixon et al., 2009
Milking frequency	Number of milkings per day	NR	0.05 (0.01)	Wethal and Heringstad, 2019
Milking interval	Difference between the begin time for the present milking and the begin time for the previous milking	SR	0.09 (0.03)–0.23 (0.05)	Carlström et al., 2013
Milking interval	Time between milking sessions	NR	0.02 (<0.01)	Wethal and Heringstad, 2019
Milking interval (hours)	Difference between the begin time for the present milking and the begin time for the previous milking	HO	0.17 (0.05)–0.26 (0.05)	Carlström et al., 2013
Number of milkings	Number of milkings per cow per 24 h	HO, SR	0.02 (0.01)–0.07 (0.01)	Carlström et al., 2013
Rear teat distance	Distance between left and right rear teat ends in mm	HO	0.37 (0.03)–0.47 (0.02)	Poppe et al., 2019
Rejected milkings	Number of visits for cows in the AMS without being milked	NR	0.02 (<0.01)	Wethal and Heringstad, 2019
Teat not found	Defined as the number of daily milkings in which the AMS was unable to find at least one of the teats for milking	NR	0.002 (0.004)	Wethal and Heringstad, 2019
Total milk yield per day	Milk yield measured by the AMS	HO	0.18 (0.06)	Viviana Santos et al., 2018
Udder balance (mm)	Average difference in distance to the floor between the front and rear teat ends	HO	0.38 (0.03)–0.40 (0.02)	Poppe et al., 2019
Udder depth (mm)	Average distance of teat ends to the floor	HO	0.65 (0.02)–0.69 (0.02)	Poppe et al., 2019

**TABLE 3:** Heritability estimates for traits derived from automated feeding systems in sheep, swine and cattle.

Trait	Measurement protocol or trait definition (general observations)	Species	Heritability (SE)	References
Feed intake at the visit	Feed intake recorded by the automatic feeder	Sheep	0.38 (0.07)	Marie-Etancelin et al., 2019
Feeding duration at the visit	Time recorded by the automatic feeder	Sheep	0.28 (0.06)	Marie-Etancelin et al., 2019
Between−visit time	Between−visit time interval	Sheep	0.38 (0.07)	Marie-Etancelin et al., 2019
Feeding rate	Defined as the ratio between feed intake and feeding duration	Sheep	0.37 (0.06)	Marie-Etancelin et al., 2019
Number of meals per day	A minimum of two consecutive data points were required to constitute a meal	Swine	0.315 (0.075)	Rohrer et al., 2013
Average meal length (s)	Meal length was calculated as number of consecutive data points times 20 s	Swine	0.604 (0.087)	Rohrer et al., 2013
Daily meal time (m)	Recorded by the feeder – no details presented	Swine	0.37 (0.079)	Rohrer et al., 2013
Percentage of meals alone	Recorded by the feeder – no details presented	Swine	0.391 (0.076)	Rohrer et al., 2013
Average number of pigs at feeder	Recorded by the feeder – no details presented	Swine	0.514 (0.081)	Rohrer et al., 2013
Percentage of meals at gate-side	Recorded by the feeder – no details presented	Swine	0.157 (0.056)	Rohrer et al., 2013
Percentage of meals at open-side	Recorded by the feeder – no details presented	Swine	0.213 (0.070)	Rohrer et al., 2013
Feeding duration, min⋅d-1	Recorded by the feeder – no details presented	Cattle	0.25 (0.16)	Durunna et al., 2011
Head-down time, min⋅d-1	Recorded by the feeder – no details presented	Cattle	0.14 (0.15)	Durunna et al., 2011
Feeding rate, kg⋅h-1	Recorded by the feeder – no details presented	Cattle	0.35 (0.16)	Durunna et al., 2011
Feeding frequency, visits⋅d-1	Recorded by the feeder – no details presented	Cattle	0.56 (0.19)	Durunna et al., 2011
Feeding duration, min⋅d-1	Recorded by the feeder – no details presented	Cattle	0.14 (0.11)	Durunna et al., 2011
Head-down time, min⋅d-1	Recorded by the feeder – no details presented	Cattle	0.09 (0.1)	Durunna et al., 2011
Feeding rate, kg⋅h-1	Recorded by the feeder – no details presented	Cattle	0.67 (0.19)	Durunna et al., 2011
Feeding frequency, visits⋅d-1	Recorded by the feeder – no details presented	Cattle	0.59 (0.18)	Durunna et al., 2011

**TABLE 4:** Heritability estimates for traits derived from various technologies and biomarkers in chicken and cattle.

Technology (summary)	Trait	Measurement protocol or trait definition (general observations)	Species	Heritability (SE)	References
Camera	Frequency of feeding	Determined by the focal sampling on each individual (a meal was defined as a sequence during which birds were continuously feeding and which was separated from another feeding event by more than 3 s)	Chicken	0.06 (0.02)	Mignon-Grasteau et al., 2017
Camera	Frequency of moving	Recorded by camera – no details presented	Chicken	0.09 (0.07)	Mignon-Grasteau et al., 2017
Camera	Frequency of lying	Recorded by camera – no details presented	Chicken	0.10 (0.06)	Mignon-Grasteau et al., 2017
Infrared sensors	Flight speed (m/s)	Infrared sensors were used to trigger the start and stop of the timing system	Cattle	0.49 (0.18)	Nkrumah et al., 2007
Milk infrared spectra	Blood β-hydroxybutyrate at 11 to 30 DIM	Blood BHB was predicted from milk spectra	Cattle	0.248 (0.005)	Belay et al., 2017
Milk infrared spectra	Blood β-hydroxybutyrate at 31 to 60 DIM	Blood BHB was predicted from milk spectra	Cattle	0.274 (0.004)	Belay et al., 2017
Milk infrared spectra	Blood β-hydroxybutyrate at 61 to 90 DIM	Blood BHB was predicted from milk spectra	Cattle	0.322 (0.005)	Belay et al., 2017
Milk infrared spectra	Blood β-hydroxybutyrate at 91 to 120 DIM	Blood BHB was predicted from milk spectra	Cattle	0.360 (0.005)	Belay et al., 2017
Transponder	Daily feeding duration (min/d)	Daily feeding duration was computed as the sum of the difference between feeding event end-times and start times per day for each animal	Cattle	0.28 (0.12)	Nkrumah et al., 2007
Transponder	Daily feeding head down time (min/d)	Sum of the number of times the electronic identification (transponder) of the animal was detected by the Growsafe system during a feeding event multiplied by the scanning time of the system	Cattle	0.33 (0.12)	Nkrumah et al., 2007
Transponder	Daily feeding frequency (events/d)	Number of independent feeding events for a particular animal in a day (recorded by the transponder)	Cattle	0.38 (0.13)	Nkrumah et al., 2007

In brief, genomic selection (Meuwissen et al., 2001) refers to the use of a large number of markers distributed across the whole genome to estimate the breeding values (and future performance) of breeding individuals for traits of interest (e.g., temperament, feather pecking). Genomics provides a great venue for genetically improving animal welfare, as it permits increasing the accuracy of breeding values for selection candidates or close relatives, even if they are not exposed to additional stressors. In this regard, data collection can be performed in chosen herds or flocks (e.g., nucleus or phenotyping herds) that are genetically connected to the potential breeding animals. This creates an opportunity to measure a large number of traits (deep phenotyping) in the same group of animals and use this information to genetically select non-phenotyped animals in commercial farms. As long as there is a sufficiently large training population (individuals with both phenotypes and genotypes) genetically related to the selection candidates, the accuracy of genomic breeding values can be moderate to high. Therefore, genomic tools facilitates selection for complex behavioral and welfare traits in commercial farms (Rodenburg and Turner, 2012). This is very advantageous, especially in the case of disease resilience, where a disease challenge might be required and cannot be performed in the nucleus farms (Putz et al., 2019).

A limited number of livestock breeding programs have included welfare indicator traits in their selection schemes (Miglior et al., 2017; Preisinger, 2018; Turner et al., 2018; Chang et al., 2020). A major challenge for the implementation of genetic evaluation for welfare traits has been the difficulty in collecting individual measurements on a large number of animals (Houle et al., 2010; Turner et al., 2018). As welfare is a multifactorial state, there is a need for simultaneously measuring multiple variables over time (repeated records). This requirement can be a major constraint in commercial breeding programs due to the infrastructure needed to collect the data, economic feasibility, standardization of data collection protocols, and lack (or reduced availability) of equipment and procedures that maximize the welfare of the animals during the measurements.

More recently, precision livestock farming (PLF) technologies (Friggens and Thorup, 2015; Berckmans, 2017), also termed digital agriculture (Liakos et al., 2018), have been presented as an alternative to individually assessing welfare indicator traits on commercial farms. These technologies rely on continuous automatic real-time monitoring and controlling of animal activities and environmental conditions (Berckmans, 2014). This is usually done using sensors (e.g., accelerometers, ruminal boluses, biosensors, and radio-frequency identification – RFID-enabled ear tags), imaging (e.g., cameras), sounds (e.g., microphones), and recording of movements (Lohölter et al., 2013; Andriamandroso et al., 2016; Terrasson et al., 2016; Neethirajan, 2017; Vranken and Berckmans, 2017; Rufener et al., 2018; Ellen et al., 2019; Halachmi et al., 2019). However, many of these technologies measure phenotypes at flock or herd level, down to pen level, with individual-level data options only more widespread for large livestock species kept in smaller numbers. In addition to PLF technologies, variables based on simpler equipment and protocols can also be collected in large scale and used to assess animal welfare (e.g., lesion scoring, hoof health scoring, docility scoring, and milking temperament assessed by animal handlers). Furthermore, computational and data science fields (e.g., machine learning, computer vision, and cyber-physical systems) are quickly advancing (Nayeri et al., 2019; Tomisław et al., 2019; Verma et al., 2020). Thus, datasets generated from PLF technologies coupled with data science developments are paramount to translate animal welfare indicators into accurate genomic breeding values to be used for selective breeding aiming to enhance animal welfare.

Previous reviews have focused on the use of precision technologies for a variety of purposes, especially on-farm management (Neethirajan, 2017; Neethirajan et al., 2017; Vranken and Berckmans, 2017; Croney et al., 2018b; Benjamin and Yik, 2019; Halachmi et al., 2019). The current review, expands this scope by focusing on the use of precision technologies for selective breeding to enhance animal welfare in commercial livestock production, with a focus on terrestrial species. In this context, our main objectives are to: (1) describe ways to develop novel welfare indicator traits (using ideal phenotypes) for both genetic and genomic selection schemes; (2) summarize key indicator variables of livestock behavior and welfare, including a detailed assessment of thermal stress in livestock; (3) describe the primary statistical and bioinformatic methods available for large-scale data analyses of animal welfare; and (4) identify major advancements, challenges, and opportunities to generate high-throughput and large-scale datasets to enable genetic or genomic selection for enhanced welfare in livestock.

## Main Requirements for Identifying Welfare Traits for Selective Breeding Purposes

Animal welfare science is a relatively new field that is quickly evolving in an interdisciplinary manner (Carenzi and Verga, 2009; Broom, 2011; Marchant-Forde, 2015). The longitudinal measurement or quantification of multiple welfare indicators is the main requirement for selective breeding to enhance animal welfare. In this section we present some ideas toward the identification and description of ideal phenotypes for selective breeding.

A phenotype, or phenotypic trait, is defined here as a variable that can be measured on a continuous (e.g., cortisol level, body temperature), or categorical (e.g., docility and longevity scores) scale in individual animals and represents a biological mechanism at a certain time point (or life stage). Animal welfare is a multidimensional concept comprising physical, behavioral, physiological, and emotional aspects (Broom, 1991; Rushen et al., 2011), and thus, its objective measurement [automated assessment with no bias or dependence on the device used (or technician doing the assessment)] is a challenging task.

Firstly, continuous monitoring of the animal welfare state from birth to slaughter (or involuntary culling) is needed because animals can be more or less prone to certain welfare issues at specific life stages [e.g., food allergies and gut inflammation after weaning in piglets (Jayaraman and Nyachoti, 2017; Radcliffe et al., 2019), tail biting and aggressive behaviors after mixing pigs in larger groups (Camerlink et al., 2013; Shen et al., 2019), feather pecking in laying hens (Ellen et al., 2019), and age-specific disease occurrences such as mastitis in dairy species (Barkema et al., 2015)]. Therefore, longitudinal phenotypes need to be collected and analyzed (Rauw and Gomez-Raya, 2015; Berghof et al., 2019; Oliveira et al., 2019a). Resilience, defined as the capacity of an animal to be minimally affected by disturbances or to rapidly return to the state attained before exposure to a disturbance (Berghof et al., 2019), can also indicate welfare. Based on longitudinal measurements, resilience indicators may be derived based on deviations from expected production levels over a period of time (Berghof et al., 2019), or variations in automatically recorded feed intake (Putz et al., 2019). For instance, Putz et al. (2019) proposed various novel phenotypes related to disease resilience using daily feed intake data from growing pigs under a multifactorial natural disease challenge that was designed to mimic a commercial environment with high disease burden. The novel resilience phenotypes proposed by the authors were heritable and genetically correlated with mortality and treatment rate (Putz et al., 2019). In the context of longitudinal measurements, it is worth noting that stress responses can be beneficial in helping the animals to cope with their environment and challenging situations. However, overstimulated stress response (too frequent or for too long) can detrimentally affect biological functions such as production, immune response, and coping abilities (Moberg, 2009; Palme, 2012; Rauw et al., 2017).

Secondly, a large number of variables need to be accurately measured in individual animals as biological indicators of the Five Freedoms (Brambell, 1965; McCulloch, 2013), including physiological, behavioral, emotional state, and physical and health characteristics. A single stressor can impact biological functions of the animal in different ways [e.g., feed deprivation can cause weight loss, hunger and frustration, behavioral changes, altered metabolic rate (Ketterson and King, 1977), and immune suppression; thermal stress can cause altered feed intake, digestion, discomfort, uneven growth and body weight, and altered metabolic function leading to distress and increased mortality (Johnson, 2018); and social isolation, group mixing and restraint can result in altered heart rate, elevated cortisol levels, frustration, aggressive behavior, and weaker immune systems (Ruis et al., 2001; Shen et al., 2019)]. Interestingly, the stress response to possible threatening stimuli varies among individuals dependent on how the stress is perceived (i.e., individual susceptibility), resulting in different individual welfare outcomes. Animals are capable of experiencing positive and negative emotions, and welfare indicators should not only focus on physical conditions but on their emotional states as well (Reimert et al., 2013; Wemelsfelder and Mullan, 2014; Jirkof et al., 2019; Lawrence et al., 2019). In addition to physiological indicators of stress, recording the prevalence of behavioral signs associated with negative welfare such as arousal and hyperactivity, frustration, distress, and depression can provide important clues about how animals are coping with their environment as well as their welfare (Keeling et al., 2011).

Thirdly, data collection should be based on non-invasive methods that do not result in additional stress or discomfort to the animals or alter their routine or circadian rhythms. For instance, handling animals for measuring blood parameters could cause stress hormone release (Stewart et al., 2005; Cook, 2012). This could be an issue for assessing the undisturbed welfare status of the animal in commercial production settings. Please note that the effect of handling-induced cortisol release can be minimized by recording the time from start of handling to end of blood collection and including it as a covariate in the models; or alternatively, training the animals to habituate to the blood collection procedure, depending on the study goals. Similarly, phenotyping animals during a stressful event intrinsic to their management environment has been suggested to be preferred than exposing animals to an experimentally imposed stressful situation (Colditz and Hine, 2016).

The derived phenotypes need to be collected at a low cost to enable measurement of a large number of animals, which is a requirement for successful implementation of genetic and genomic evaluations (Goddard et al., 2010), as previously discussed. Obtaining phenotypic measurements that are accurate, biologically meaningful, repeatable, and comparable among laboratories, countries, or companies, is critical for genomic studies and its applications (Hocquette et al., 2012). Therefore, standardizing measurement protocols or defining phenotypes that can be easily standardized is needed because traits recorded in different ways might reflect different biological mechanisms, which may lead to difficulty in the implementation of genetic and genomic evaluations based on datasets from multiple phenotyping centers (or farms, countries, etc.). This is still challenging as there are not enough welfare studies to support differences in such protocols. The lack of available datasets and optimal protocols indicates a need for worldwide funding agencies (private and public) to increase financial support for phenotyping animal welfare indicators for breeding purposes. This has been recently included as a key priority in some agricultural funding agencies as outlined in the latest USDA Blueprint for Animal Genome Research 2018–2027 (Rexroad et al., 2019).

Lastly and critically important, the phenotypes identified need to be heritable and repeatable. Low heritability might only indicate high phenotypic variability in comparison to the total additive genetic variance. Therefore, when necessary, it is crucial to identify alternative variables that can better capture the genetic variability for the trait(s) of interest (i.e., higher heritability; König and May, 2019). The rate of genetic progress for a certain trait also depends on the generation interval (Falconer and Mackay, 1996), and therefore, traits that are measured earlier in life, but reflect the welfare status of the animal in its whole life (or at a later stage), are desirable. In this context, genomic selection is a very powerful tool, as it enables the calculation of genomic breeding values for young animals with no phenotypic measurements (i.e., reduce generation interval). The genetic correlation between welfare and commonly selected traits also need to be investigated and appropriately weighted in selection indexes to avoid detrimental effects in other important traits (Phocas et al., 2016a, b).

The greater availability of high-throughput phenotyping technologies (e.g., automated monitoring systems) in nucleus and commercial farms, better communication and data sharing among data recording organizations (e.g., Dairy Herd Improvement, breed associations, veterinary clinics, and slaughter facilities), and greater integration of complementary disciplines will contribute to overcoming some of the challenges associated with time and cost of welfare data collection (Wemelsfelder and Mullan, 2014). In addition, PLF tools enable the collection of continuous and real-time phenotypes as well as environmental conditions (e.g., thermal stress, humidity, air quality; Laberge and Rousseau, 2017), that are of great use for assessing animal welfare.

## Welfare Assessment in Livestock Production

The welfare of animals is determined by the interaction between intrinsic animal characteristics and the environments in which they are raised. The definition of welfare indicators is largely dependent on a clear understanding of the biological and emotional mechanisms behind the phenotypic variability observed in the animal’s response to different stimuli. Novel indicators are being proposed as the animal welfare science moves forward. As discussed by Marchant-Forde (2015), accurate welfare assessment should be comprised of components that describe or quantify cellular, physical, physiological/biochemical, and psychological states, and may include scoring scales for additional health and behavior indicators such as body weight, respiration rate, ocular discharge, feces condition, and provoked behavioral response (Marchant-Forde, 2015). Vertical phenotyping is therefore of great importance because several variables can be related to a family of phenotypic traits (Hocquette et al., 2012).

The aggregation of multiple indicators to produce an overall assessment of animal welfare is of great relevance (Botreau et al., 2007a, b). One can expect that genomic selection for improved welfare will continue to be a very interdisciplinary field, integrating animal welfare, cell and molecular biology, neuroscience, immunology, stress physiology, computer science, engineering, quantitative genomics, and bioinformatics. This section will succinctly review biological mechanisms behind animal welfare and how this knowledge can be used for the identification of novel welfare indicators for breeding purposes.

### Biological Mechanisms Related to Animal Welfare

Livestock in commercial production systems are constantly exposed to a variety of environmental stressors or management practices (e.g., human presence, noise, strange objects, restricted space, heat, cold, humidity, and feed restriction). Therefore, the animals’ welfare, productivity, and environmental fitness will rely on their ability to cope with and react to these challenges (Broom, 1991; Guy et al., 2012; Colditz and Hine, 2016; Berghof et al., 2019; Hu et al., 2019). At any point in time in which an animal is exposed to a variety of potential challenges or stressors, it will counteract using behavioral and physiological processes or sub-systems, linked through a network of neural and hormonal communication. The stressors may vary in magnitude and duration – being short-term (acute) or longer-term (chronic) – and if the animal’s processes counteract and adjust successfully, the animal copes with the stressor and habituates (Moberg and Mench, 2000). This ability to cope and habituate is the cornerstone of resilience – the ability to use these biological sub-systems to bounce back to “normal functioning” after disturbance (Scheffer et al., 2018). An animal with high resilience is able to recover quickly from larger disturbances and there is low temporal autocorrelation in the fluctuations of any given sub-system working to counteract disturbances (Scheffer et al., 2018; Berghof et al., 2019). There is also the ability of the sub-systems to work more independently in animals with high resilience and to return the animal to the baseline state, before its interconnected sub-systems are also activated. With low resilience, the opposite is true. A small disturbance may show a slow recovery, high temporal autocorrelation, and high inter-dependence among sub-systems, with the worst-case scenario resulting in a cascade of sub-system failure (Scheffer et al., 2018).

Within animal agriculture, the main causes of stress include environmental, immunological, metabolic, and social factors. Some may be acute, for example, a single aggressive interaction after mixing which is quickly resolved; some may be chronic, for example, periods of sustained heat during summer months; and some may even be permanent. A stress response is activated when the central nervous system perceives a potential threat to homeostasis. From the central nervous system, electrochemical impulses are transmitted to the effector organs of the body (muscles and glands) to initiate appropriate responses to the stimuli (Cheng, 2010). The defense response consists of a combination of four general biological responses (Moberg and Mench, 2000): the autonomic nervous system response, the neuroendocrine response, the immune response, and the behavioral responses. Under extensive conditions, behavior can often be adapted to mitigate the stress quickly. If confronted by aggression, an individual can retreat and end the encounter if given enough space. If hot, the animal can seek shade or wallow. In farming systems, the behavioral processes may be more constrained, and lack of space or thermal zones can mean that an immediate behavioral response is not possible, as in these two examples. The individual’s response to external stressors can be influenced by numerous factors including prior experience, genetics, age, sex, physiological status, emotional state, and cognitive ability (Colditz and Hine, 2016).

The intricate details of stress system activation are available elsewhere (Godoy et al., 2018), but generally, both physical and psychological stressors interact through different pathways to activate the hypothalamic-pituitary-adrenal (HPA), and sympathetic-adrenal medullary (SAM) systems, which activate together multiple sub-systems to maintain homeostasis. The SAM axis results in the release of catecholamines, such as epinephrine (E) and norepinephrine (NE), from the adrenal medulla. The concentrations of E and NE are increased due to a variety of stressors (Dalin et al., 1993) and activation is rapid, within one to two seconds, since E and NE half-lives are short. Simultaneous to the activation of the SAM axis, the hypothalamus also activates the HPA axis releasing corticotropin-releasing factor from the paraventricular nucleus of the hypothalamus. Corticotropin-releasing factor stimulates the anterior pituitary to release adrenocorticotropic hormone which activates the adrenal gland to secrete glucocorticoids (i.e., cortisol, corticosterone) into the blood. Therefore, cortisol concentrations have been used as an indicator of stress (Ott et al., 2014), but not without debate as to the appropriateness and need for refinement (Ralph and Tilbrook, 2016). Glucocorticoid release is much slower than the release of catecholamines, in most species beginning around 2 min after the stressor. However, there is also a circadian pattern to glucocorticoid release due to their priming effect and thus, there are limitations in relying on single time-point samples. The glucocorticoids act collectively with the catecholamines to increase blood glucose (Dallman and Hellhammer, 2011), thus ensuring that there are enough energy reserves needed to mitigate the stressors. Furthermore, the release of cortisol elicits a negative feedback response to the HPA axis to return to basal levels and homeostasis (Manteuffel, 2002; Stephens and Wand, 2012). There is large variation in the response of the various components of the HPA axis (Mormède et al., 2011), indicating a clear potential to genetically select for biological changes in the stress response.

### Indicators of Animal Welfare

There is large variability in animal’s response to stress factors (Turner, 2011; Koknaroglu and Akunal, 2013; Turner et al., 2018). Therefore, welfare assessment is needed in order to identify the most resilient and healthiest animals for breeding purposes as well as to develop mitigation strategies to minimize or eliminate welfare issues. The evaluation of animal welfare involves a complete assessment of the animal’s physiological, behavioral, physical, and emotional state. Some of these indicators can even be quantified prior to clinical signs of poor welfare (e.g., milk somatic cell count and clinical mastitis). This complete assessment relies on some key principles, such as those developed in the Welfare Quality Project (described in Rushen et al., 2011): good feeding, proper housing, good health conditions, and appropriate behavior. These conditions can be assessed based on various parameters, including aggressive behavior when mixing or regrouping animals [especially in pigs (Wurtz et al., 2017; Shen et al., 2019)], approach or avoidance behaviors (Smulders et al., 2006), blood parameters (König and May, 2019), body condition score (Roche et al., 2009), body mutilations [e.g., tail damage (Keeling et al., 1996; Heinonen et al., 2010)], body temperature (Weschenfelder et al., 2012), cannibalism (Lambton et al., 2015), feather pecking (Buitenhuis et al., 2003), feeding behavior [e.g., active chewing time, rumination time, standing and lying time (Ding et al., 2018)], proportion of time active and its posture (Vasseur et al., 2012), immune response (Kovács et al., 2014), response to infection (Nyman et al., 2014), inflammation (Heinonen et al., 2010), heart and respiration rates (von Borell et al., 2007), glucocorticoids (corticosterone and cortisol; Mormède et al., 2011; König and May, 2019), lameness and gait problems (Chapinal et al., 2013), panting frequency (Sullivan et al., 2011), poor maternal care [e.g., savaging in pigs (Hellbrügge et al., 2008b)], ruminal pH (indicator of digestive issues, such as ruminal acidosis; Abdela, 2016), shivering (Liu et al., 2017), social interactions (Rault et al., 2013), abnormal repetitive behaviors (Mason, 2006; Olsson et al., 2011), frustration behaviors (Duncan, 1998; Keeling et al., 2011), variations in daily feed intake (Putz et al., 2019), and water intake (Kume et al., 2010). As previously mentioned, this large number of variables indicates that overall animal welfare needs to be assessed based on a combination of multiple traits.

### An Example of Welfare Assessment: Quantifying Thermal Stress in Livestock

**FIGURE 1:**
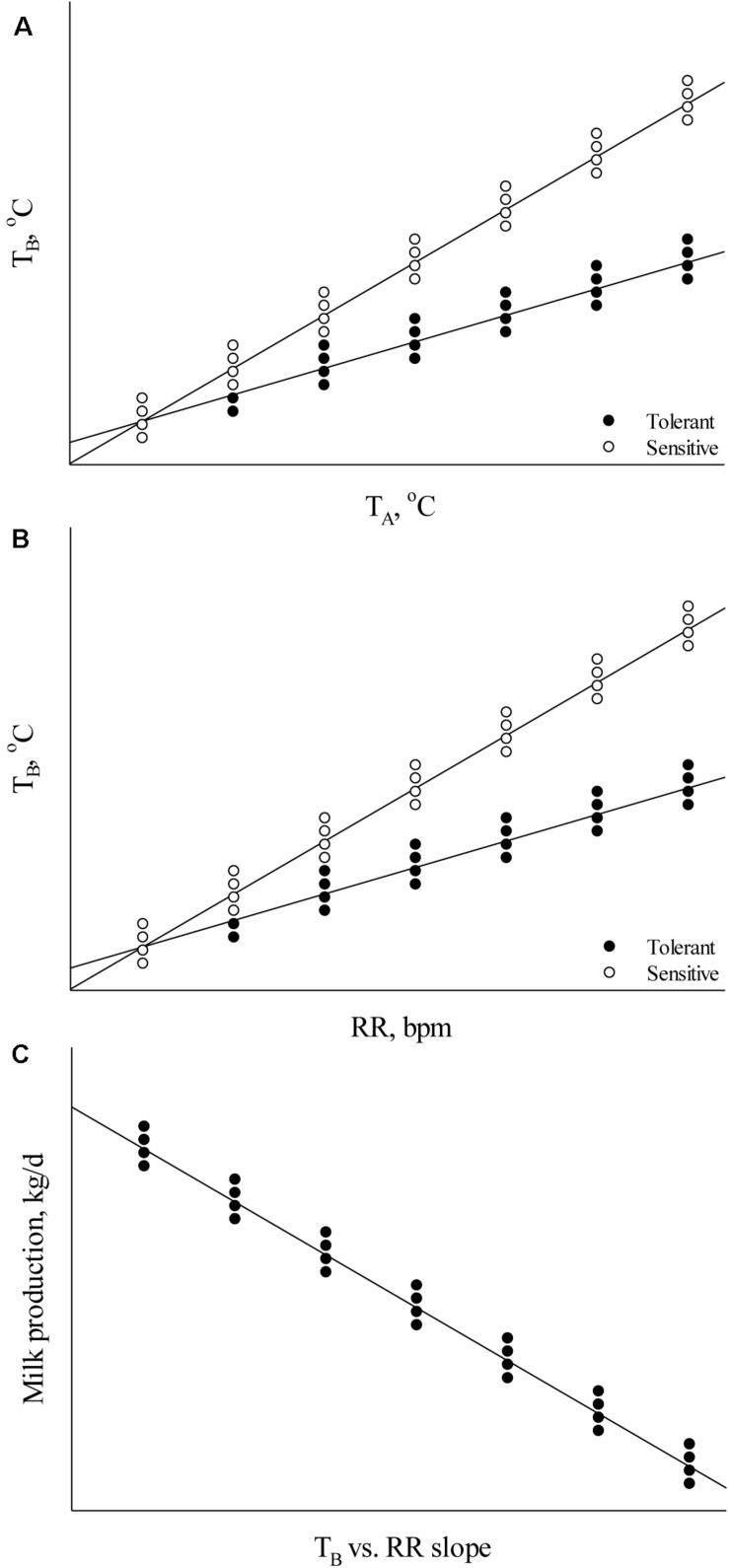
Relationships between **(A)** body temperature (T_B_) and ambient temperature (T_A_), **(B)** T_B_ and respiration rate (RR), and **(C)** milk production and the T_B_ vs. RR slope.

Body temperature measurements facilitate determination of the animal’s thermoregulatory ability under varying environmental conditions. These phenotypic records may be valuable in selecting breeding stock with improved welfare under environmental conditions that cause heat stress (Carabaño et al., 2017, 2019). Heat tolerance is heritable (Table 1; Ansari-Mahyari et al., 2019; Carabaño et al., 2019; Osei-Amponsah et al., 2019) and causes major welfare and economic losses to the livestock industry (Mayorga et al., 2020); however, the ability to appropriately analyze and understand phenotypic indicators is necessary for the development of new breeding programs to select for heat tolerant animals. Absolute body temperature (T_B_) measures may be used to assess an animal’s heat stress response whereby greater T_B_ can indicate increased heat sensitivity and reduced T_B_ can indicate greater heat tolerance (Johnson, 2018). For the simplest analyses, either daily average T_B_ or T_B_ during certain time periods (e.g., morning, afternoon, and night-time) may be calculated to compare between animals under differing environmental heat loads. As an assessment of T_B_ responsiveness, the T_B_ change rate as a function of increasing heat load (Figure 1A) can be used to determine heat stress sensitivity. In addition, these data can be used to determine the ability of animals to acclimate or adapt if compared across heat stress exposure days, whereby a greater decrease in T_B_ responsiveness over exposure days can indicate improved acclimation ability and these data may be important markers for selecting animals with better heat stress coping abilities.

Although these analyses are valuable in initial thermal sensitivity assessments, these data alone cannot explain the underlying cause of thermal sensitivity or tolerance. This is important when trying to balance heat tolerance with maintained productivity because heat tolerance may be an outcome of decreased metabolic rate resulting from decreased performance (Brown-Brandl et al., 2014), which is not a desirable outcome under commercial production conditions. Therefore, understanding how animals dissipate excess body heat and how heat dissipation interacts with heat tolerance and productivity is an important factor to consider in breeding programs. When obtaining phenotypic thermoregulatory data, it is important that measures of heat dissipation (e.g., respiration rate – RR, skin temperature – T_S_, and sweating rate – SR) are taken in combination with T_B_ to ascertain information about an animal’s capacity to maintain euthermia as heat dissipation influences T_B_, and T_B_ influences heat dissipation (Blatteis, 1998). Balancing heat production with heat loss is essential under environmental conditions that cause heat stress in animals. Animals with improved performance (e.g., milk production, growth rate, and egg production) generate greater metabolic heat when compared to their lower producing counterparts (Brown-Brandl et al., 2014; Cabezón et al., 2017). In turn, the heat sensitivity of high producing animals may be increased if heat dissipation capacity is not sufficient.

Several analyses may be used to assess relationships between heat dissipation mechanisms and T_B_. To determine heat dissipation efficiency through the skin, the relationship between T_S_ and T_B_ can be calculated. As heat dissipation through the skin is reliant on core T_B_, an increased ratio may indicate greater heat dissipation. However, this ratio may be influenced by the external environment (e.g., cooler temperatures cause vasoconstriction and warmer temperatures cause vasodilation; Blatteis, 1998) and thus ambient temperature can be used in the analysis. In this case, the thermal circulation index may be calculated using T_S_, ambient temperature, and T_B_ as described by Curtis (1983): thermal circulation index = (T_S_ – ambient temperature)/(T_B_ – T_S_). The thermal circulation index can be used to determine the capacity of an animal to dissipate heat from the core to the skin and subsequently to the surroundings under steady state thermal conditions (Kpodo et al., 2019). In addition to T_S_, the assessment of T_B_ as a function of RR may be used to assess RR efficiency whereby a greater T_B_ slope with increasing RR indicates reduced RR efficiency and a decreased slope indicates increased efficiency (Figure 1B). This is an important factor to consider outside of absolute RR values because an increase in RR may not necessarily indicate greater heat sensitivity if the end result is a euthermic T_B_. Alternatively, comparing RR as a function of T_B_ may explain heat sensitivity in which a lower RR rise with increasing T_B_ can explain heat sensitivity if the RR increase is not sufficient to dissipate excess body heat. These methods may also be applied to the assessment of SR. Finally, results from these thermoregulation analyses may also be compared with performance parameters to determine their influence on growth rate, reproductive success, milk output, and egg production (Figure 1C). These data can enable balancing improved welfare under heat stress conditions with performance measures and evaluate which thermoregulatory measure is most important in a particular system.

There are multiple strategies for increasing heat tolerance, such as within-breed genetic or genomic selection (Nguyen et al., 2017; Carabaño et al., 2019), crossbreeding or the use of more climatic adapted genetic resources such as Zebu cattle (*Bos taurus indicus*), and slow growing or less-feathered birds (Singh et al., 2001; N’dri et al., 2007; Fathi et al., 2013). Furthermore, gene editing might also be an important tool for introgressing certain gene alleles that confer greater heat tolerance (Hansen, 2020), such as the “slick hair” gene in cattle (Littlejohn et al., 2014), and “naked neck” and “frizzle” genes in chicken (Fathi et al., 2013).

#### Phenotyping Technologies Used to Assess Thermal Stress

Body temperature measures are commonly used to assess the thermoregulatory capacity of animals. These measures often include RR, SR, T_S_, and T_B_, and these phenotypic traits are most commonly used as a determination of heat stress. Traditionally, these measures were obtained through labor intensive and invasive practices. However, in recent years, several non-invasive and/or automated methods to collect these data have been developed.

##### Skin temperature

During heat stress, blood flow to the skin increases to facilitate heat dissipation, which may be measured by an increase in T_S_ (Yahav et al., 2005; Katiyatiya et al., 2017). However, environmental factors such as wind speed, humidity, and direct sunlight exposure (Church et al., 2014), or physical factors such as hair thickness, hair length, and hair and skin color (Gebremedhin et al., 2008) can impact the efficiency of heat loss through the skin or directly alter the T_S_ independent of changes in T_B_ (i.e., direct sunlight exposure, exposure to heating elements, etc.). Interpreting T_S_ values requires additional inputs and considerations. For animals housed outdoors without shade (i.e., cattle on pasture or in feedlots) or under heating elements (i.e., pigs or chickens under heat lamps), it is difficult to separate the effects of the environment on changing T_S_ compared to the influence of T_B_ on increasing/decreasing T_S_ due to heat dissipation through the skin. It is important to consider that T_S_ measures greater than T_B_ should not be interpreted as heat dissipation as it is impossible to dissipate a greater amount of heat than is produced within the body and it is likely that these values are indicative of environmental influences on the T_S_ rather than changing T_B_. In cases where radiant heat is not a factor (i.e., environmental chambers, in the shade, etc.) T_S_ measures (on shaved or hairless skin) may be helpful in the assessment of heat dissipation for the selection of more heat tolerant animals and a common, non-invasive method to assess T_S_ is through infrared thermography (Ferreira et al., 2011; Nääs et al., 2014; Lees et al., 2018). Taken together, researchers must consider these factors when making determinations about the significance of changing T_S_ in relation to heat dissipation vs. radiant heat load.

Infrared thermography measures the infrared radiation emitted from an animal and this radiation depends on the temperature, emissivity, and conductivity of the animal (Knízková et al., 2007). There are two types of infrared systems to measure temperature on animals: infrared thermometers and thermal cameras. Infrared cameras are more software intensive than infrared thermometers and can be used for monitoring large areas (Sellier et al., 2014), which allow for a greater representation of the T_S_ of the entire animal or at specific sites as desired by the researcher. An alternative to infrared technology that may be more invasive are contact sensors affixed to the skin (Teunissen et al., 2011; Mostaço et al., 2015). Contact sensors are more accurate than infrared technology and provide continuous automated measurements, but potential issues precluding their use may include battery life and long-term adhesion to the skin (Mostaço et al., 2015), and destruction or loss of the devices in group-housed animals. Therefore, researchers should assess both types of technology and determine which one best fits their requirements in a particular environment or research setting.

##### Respiration rate

In general, animals cope with heat stress by increasing RR to reduce the extra heat load via evaporative heat loss. However, it is important to mention that during extreme heat strain when heat loss cannot be balanced with heat gain, animals will switch from increased RR to deep slow respirations (López Armengol et al., 2017). One way to measure RR is visually by counting flank movements at the flank region (Mostaço et al., 2015). While this traditional method is regularly used, it is labor-intensive and time consuming. As an alternative to this method, researchers have developed technologies that assess RR through changes in air temperature near the nostrils of animals using infrared thermography (Lowe et al., 2019), or direct measures of air temperature near the nostrils using a mounted device (Milan et al., 2016). The use of sensors to detect nasal exhalation pressure has been proposed to evaluate RR in cattle (Strutzke et al., 2019). Finally, researchers have also used an externally-mounted bioharness designed for humans, that measures chest expansion (Briefer et al., 2015). Unfortunately, many of the automated methods to assess RR are in development and there are currently no known commercially-available and validated options for researchers to automatically (and non-invasively) assess RR in livestock animals.

##### Sweating rate

Cattle increase SR to dissipate excess body heat through evaporative heat loss from the skin surface. Heat loss via sweating may be influenced by wind velocity, air temperature, relative humidity, and thermal and solar radiation (Collier and Gebremedhin, 2015). The SR can be determined using a digital moisture sensor on the dorsal areas of animals to determine *trans*-epidermal water loss (Nuutinen et al., 2003; Gebremedhin et al., 2008). The digital moisture sensor is a closed system, free of ambient airflow, and allows for monitoring of water loss (Scharf et al., 2008). Another method to measure SR is applying a cobalt chloride disk to the skin and recording the length of time the cobalt chloride disk changes color in order to calculate SR (Moser et al., 2012; Nursita and Cholis, 2019). However, a potential drawback to this method in animals is the ability to maintain the disk on the skin for the length of time required for the color change to occur.

##### Body temperature

Heat stress causes an increase in T_B_ implying that the animal has lost the ability to maintain homeostasis. In pigs, infrared (Mostaço et al., 2015), and digital clinical thermometers (Gebremedhin et al., 2008; Mostaço et al., 2015) are commonly used to measure T_B_ rectally. However, when using a clinical thermometer, restraint is often required, which can stress the animal and potentially increase T_B_. Other reliable and accurate T_B_ measurement devices include surgically implanted telemetry devices (Lacey et al., 2000) and intramuscularly implanted microchips (Iyasere et al., 2017). Both devices are good for automatically collecting data at pre-set intervals, but have the risk of infection after surgery and a greater recovery time prior to data collection. In cattle, less invasive studies have used automatic measurements of reticule-rumen boluses (Timsit et al., 2011; Liang et al., 2013), which give continuous rumen temperature measurements in real time (Lohölter et al., 2013; Lees et al., 2018). In pigs, gastrointestinal temperature can be measured using orally administered temperature sensors (or boluses, as commonly defined in similar sensors used in cattle studies) monitored with a wireless core body temperature data recorder (Johnson et al., 2016). Although the boluses allow measurement without disturbing the animal, they have short communication distances between the bolus and reader thus requiring manual data collection, the boluses are costly, and T_B_ fluctuations may exist depending on the temperature of feed and water consumed (Lee et al., 2016b). Alternatively, vaginal implantation of wireless sensors can accurately determine T_B_ using a radio-telemetric system (Kyle et al., 1998; Johnson and Shade, 2017) or a temperature logger (Gebremedhin et al., 2008). Specifically, in pigs (Johnson et al., 2016), beef cattle (Burdick et al., 2012), and dairy cattle (Garner et al., 2016), vaginal temperature can be measured with a thermochron temperature recorder attached to a plastic device controlled internal drug releasing device. However, this is only effective in females. Finally, temperature sensing with an ear canal radiotelemetry system can be used on cattle due to its long-distance wireless communication and simple attachment similar to ear tagging (Lee et al., 2016b), which provides temperature stability but has the risk of the tagged device to fall off.

## High-Throughput Phenotyping Technologies

The rapid development of integrated biological (e.g., *-omics* technologies) and engineering systems and the Internet of Things (IoT) is enabling the development of affordable monitoring devices and high-throughput technologies (Neethirajan et al., 2017). These tools can be used for individually monitoring large numbers of animals in commercial settings and are advantageous to quantify biological indicators through rapid, repeatable, and automated measurements. This is crucial because the ideal welfare assessment indicators should be as objective as possible, robust (can be applied under a wide range of on- and off-farm situations), relevant and valid (reveal aspects of the animal’s affective or physiological state that is important to their welfare), reliable (can be repeated with confidence in the results), cost-effective, and well accepted by all industry’s stakeholders (Fleming et al., 2016).

The technological devices used include sensors such as cameras, microphones to capture vocalizations, thermometers, automated feeding and milking systems, automatic scales to measure body weight and lean-fat ratios, milk spectral data, electrodes to detect skin conductivity and heart rate, and accelerometers (Vranken and Berckmans, 2017; Benjamin and Yik, 2019; Halachmi et al., 2019). In this section, we describe phenotyping technologies that can be (or have been) used to assess animal welfare and potentially incorporated in genetic or genomic evaluation schemes in commercial livestock systems. It is important to note that some of these technologies are still under development and validation stages. In some cases, there could exist disagreements on their ability to assess welfare (de Rosa et al., 2019). We have highlighted examples from multiple species, but it is worth noting that the technologies and indicator traits described in this study can be easily translated or extrapolated from one species to another.

### Biomarkers

As previously indicated, various endocrine and behavioral mechanisms are involved in coping with stressors (e.g., aggression, hunger, and disease challenge). Glucocorticoids, secreted by the adrenal glands, are the most evident indicators of a stress response (Cook, 2012; Palme, 2012). They are usually measured in plasma samples; however, blood collection itself can cause additional stress as a result of handling and restraint (Cook, 2012). Palme (2012) discussed various non-invasive methods for the determination of glucocorticoids or their metabolites in saliva, urine, excreta, milk, hair/feathers, and eggs. Fecal and hair (or feather) samples are promising alternatives as circulating hormone levels are integrated over a certain period of time and are less affected by short fluctuations (Palme, 2012; Pawluski et al., 2017). The frequency of sample collection will depend on whether the impact of acute or chronic stress factors is being evaluated.

In addition to cortisol, various blood-based biomarkers have been associated with aggression in pigs, including plasma triiodothyronine (T3), 5-hydroxytryptamine, and tryptophan (Shen et al., 2019). Furthermore, disease challenge is another great welfare impairment. Huzzey et al. (2011) evaluated the potential of using pre-partum analytes associated with stress (cortisol) or inflammation (haptoglobin), and NEFA (non-esterified fatty acids) as indicators of increased risk for health complications after calving. The authors reported that NEFA was a more suitable post-partum health indicator compared to fecal or plasma cortisol metabolites, and plasma haptoglobin.

In some species (e.g., dairy cattle, dairy sheep, and dairy goats), additional biomarkers can be identified in body fluids measured routinely, such as milk. For instance, in milk, mid-infrared spectrometry (MIR) has been used to monitor potential metabolic issues and diseases such as mastitis, ketosis, fat–protein ratio, NEFA or phospholipids, glucose, and insulin growth factor 1 (Egger-Danner et al., 2014; Tetens et al., 2015; König and May, 2019), usually associated with negative welfare implications in production systems. In this regard, fat–protein from routine milk recording data has been indicated as a selection criterion to improve metabolic stability (Koeck et al., 2014). As such, various research projects have investigated the use of milk MIR data for prediction of novel indicator traits for selection purposes [e.g., RobustMilk, Opti-MIR, PhenoFinlait, and GplusE (Egger-Danner et al., 2014)].

Mastitis is a disease with major welfare implications in dairy species (Martin et al., 2018). Test-day somatic cell count (transformed to somatic cell score) is a routinely collected phenotype that has already been included in commercial breeding programs to improve udder health (Miglior et al., 2017; Martin et al., 2018). Minerals (e.g., Ca, K, Mg, Zn, Se, and P) or mineral content measured via milk MIR has also been suggested as potential biomarkers to improve mastitis resistance (Egger-Danner et al., 2014), and milk protein fractions as suitable biomarkers for heat tolerance (Carabaño et al., 2017).

Animals raised in extensive production systems (e.g., beef cattle, sheep) can suffer substantially from endoparasite infections caused by gastrointestinal nematodes (Papadopoulos et al., 2012). Various biomarkers have been proposed to genetically select for host resistance (i.e., ability to control pathogen burden) or tolerance (i.e., ability to limit the impact of a given pathogen burden on performance), but serum or milk antibodies (different isotypes of immunoglobulins), and fecal egg count are the most commonly used indicators (Bishop and Morris, 2007; König and May, 2019).

In ruminant species, measuring rumen pH can indicate metabolic and nutritional dysfunctions associated with negative welfare implications such as acidosis (Leek, 1983; Hamilton et al., 2019). There are various sensors available to measure H-ion concentration in the rumen by electrical means. These sensors (or boluses) are usually coupled with radio-frequency transmitters for continuous real-time data acquisition and there are already various commercially available devices (Mottram et al., 2008; Kim H. et al., 2018; Hamilton et al., 2019). Technology prices are decreasing over time and its quality is improving (e.g., robustness, battery life). Such devices can generate a large amount of data to be used for identifying disease resilient animals for breeding purposes.

There is also a potential to use biosensors for breath analysis aiming to identify disease indicators (bovine respiratory disease, tuberculosis, brucellosis, and ketoacidosis), especially volatile organic compounds (Fend et al., 2005; Burciaga-Robles et al., 2009; Neethirajan et al., 2017). Biosensors to analyze metabolites in sweat [e.g., lactate levels; indicator of physical stress (Jia et al., 2013)] have also been developed and converted to portable formats [e.g., belts, adhesive RFID sensor patch (Neethirajan et al., 2017)]. A large number of alternative compounds have been investigated over time, including adrenaline, noradrenaline, corticotropin-releasing factor, prolactin, glucose, lactic acid, blood leukocyte levels, and cellular immune response (Neethirajan et al., 2017). There are various bioanalytical devices and wearable technologies that can be implanted on the animals to analyze sweat composition [e.g., sodium and lactate content (Garcia et al., 2016; Glennon et al., 2016; Heikenfeld, 2016)], and assess body temperature (Sellier et al., 2014) such as wireless temperature sensor nodes that can be appressed to the base of calf’s tail (Nogami et al., 2014), detection of analytes and pathogens (Mungroo and Neethirajan, 2014; Vidic et al., 2017), and many others (Neethirajan, 2017).

The development of biosensors is rapidly advancing in human research (Metkar and Girigoswami, 2019), and one can expect that these technologies will soon be adapted to the livestock industry. High-throughput phenotyping of physiological and metabolic changes combined with large-scale genomic (and other *-omic*) datasets will be paramount on implementing genomic selection for improved animal welfare in commercial farms. It is important to highlight that it is very unlikely that a single or few biomarkers could be used for a holistic assessment of animal welfare. However, welfare biomarkers can be complementary to other data sources.

### Machine Vision (Cameras)

Machine vision has been used for several purposes in animal sciences, including determination of body weight (Tscharke and Banhazi, 2013; Kongsro, 2014), body condition score (Azzaro et al., 2011; Halachmi et al., 2013), detection of aggressive behavior (Lee et al., 2016a; Chen et al., 2017; Nasirahmadi et al., 2017), walking patterns and lameness (Stavrakakis et al., 2015), and posture and behavior during lactation (Lao et al., 2016). Often, video recordings are used to manually assess animal behavior (Oczak et al., 2013), but the manual analysis of these videos is time-consuming, and may introduce human error (Catarinucci et al., 2014).

A wide variety of cameras are available (e.g., RGB, infrared thermography cameras, 3D cameras), and more recently, there is an increasing number of research projects investigating the automation of machine vision and data analytics (Ventura et al., 2020). Therefore, machine vision is expected to play an important role in the design of large-scale data collection for breeding schemes to improve animal welfare. For instance, 3D cameras [e.g., Microsoft Kinect (Microsoft, Redmond, and Washington) and Intel RealSense (Intel, Portland, and Oregon)] are usually equipped with a high-definition camera, an infrared illuminator, and time-of-flight (ToF) depth sensor that produces color (Benjamin and Yik, 2019). These cameras are reasonably cost-effective, can handle large amounts of data, have low power requirements, do not require any contact with the animal (remote measuring), and are adaptable to variable light and background conditions (Benjamin and Yik, 2019).

Infrared thermography or thermal imaging is increasingly being used as a non-invasive method to assess animals’ physiological and emotional state, including skin temperature, inflammation in certain areas of the body (e.g., udder – mastitis), locomotion disorders, and respiratory diseases (Stewart et al., 2005; Alsaaod et al., 2014; Harris-Bridge et al., 2018; Jorquera-Chavez et al., 2019). Boileau et al. (2019) used infrared thermography taken from pigs in a controlled test environment and indicated that the obtained peripheral temperature provided useful information about the physiological and welfare outcomes of aggressive behavior in pigs. Moreover, image motion feature extraction was used for recognition of aggressive behaviors among group-housed pigs, with an accuracy of 95.82 and 97.04% for medium and high aggression, respectively (Chen et al., 2017). Infrared thermo-imaging has also been investigated as a potential tool to quantify the number of ticks in the body surface of Brangus cattle, which causes major health and welfare issues, especially in tropical countries (Barbedo et al., 2017).

In the swine industry, farrowing is a challenging stage for both the sow (transition from gestation to farrowing and lactation), and the piglets (susceptibility to crushing, chilling, and malnutrition; Marchant et al., 2001; Johnson and Marchant-Forde, 2009). Aiming to identify solutions to these issues, Leonard et al. (2019) monitored behavioral activities of sows and piglets in a commercial setting utilizing an autonomous machine vision system. A digital and ToF depth imaging system was implemented and a process with minimal user input to analyze the collected images was developed to calculate the hourly and daily posture and behavior activities of sows housed in individual farrowing crates. Depth sensors were placed on top of each stall in three farrowing rooms and controlled by mini-computers. Algorithms were able to accurately classify sow behavior (sitting: 99.4%, standing: 99.2%, kneeling: 99.7%, and lying: 99.9%). This autonomous system enables acquisition of a large amount of replicated data, and this research is a great example of integrated technology into on-farm environments that can potentially generate phenotypic records for genomic selection purposes. Lao et al. (2016) also used a machine vision-based system that automatically recognizes sow behavioral activities (e.g., lying, sitting, standing, kneeling, feeding, drinking, and shifting) in farrowing crates. The system consists of a low-cost 3D camera that simultaneously acquires digital and depth images and a software program that detects and identifies sow behaviors. This algorithm achieved an accuracy of 99.9% for lying, 96.4% for sitting, 99.2% for standing, 78.1% for kneeling, 97.4% for feeding, 92.7% for drinking, and 63.9% for transitioning between behaviors. As sows are individually housed in farrowing crates, these systems will likely be very useful for selective breeding for maternal ability [e.g., maternal behavior, piglet survival (Hellbrügge et al., 2008a)], and other alternative breeding goals (Baxter et al., 2011; Muns et al., 2016).

Another use of machine vision is analyzing the overall posture of the animal to detect lameness (and genetically select for improved hoof health). Blackie et al. (2013) evaluated kinematic gait analysis to assess stride characteristics, joint flexion and spine posture in dairy cows with different lameness status. The dairy cows were video-recorded walking along an alley (1.6 m wide), with colorful markers placed in specific parts of their bodies. In this case, the need for markers is a limitation for measuring large numbers of animals. Under farm conditions, body movement pattern recognition was applied to identify lameness in dairy cattle with an accuracy of 76% (Viazzi et al., 2013). Abdul Jabbar et al. (2017) used three-dimensional (3D) video data to analyze gait asymmetry by simultaneously tracking the movements of the spine and hind limbs of dairy cows and precisely identified 95.7% of lame cows. Body condition score is another variable that can be automatically recorded, including through the use of a Kinect camera (Microsoft Corp., Redmond, WA, United States) triggered by passive infrared motion detectors (Spoliansky et al., 2016), or by modeling cow body shape from digital images (Azzaro et al., 2011).

Tail biting is a major welfare issue in the swine industry and is a heritable trait [i.e., can be reduced through selective breeding (Breuer et al., 2005)]. Brünger et al. (2019) used neural networks to identify tail lesions in pictures from 13,124 pig carcasses and was able to correctly identify 74% of tail lesions and 95% of tail losses. Also in pigs, the behavioral and clinical alterations of growing pigs infected with two common strains of *Salmonella* spp. were investigated using a video-recording system (Ahmed et al., 2014). Recordings were able to detect clear changes in pigs’ movement, feeding and drinking behavior in response to *Salmonella* spp. infection. Additionally, Porto et al. (2015) used a multi-camera video-recording system to detect cow feeding behavior with an accuracy of 88% for feeding and 86% for standing behavior. Furthermore, Vetters et al. (2013) used an infrared sensor to determine the flight speed, to cross a fixed distance of 1.83 m, when exiting the squeeze chute as an indicator of cattle temperament.

Heart rate and heart rate variability are indicators of cardiovascular system functioning and cardiac autonomic modulation that are used to estimate physiological and psychological stress in animals (von Borell et al., 2007). In recent years, optical methods for measuring heart rate have received increased interest and technical development (Halachmi et al., 2019). Beiderman et al. (2014) proposed a photonic remote sensing system assembled on a robotic platform to measure important biological indicators such as heart beating, breathing and chewing activity. In this research, the algorithm development used image processing and image pattern recognition techniques. This promising technology can be used in livestock breeding farms to generate useful and practical information about animal welfare and stress resilience to incorporate into breeding programs.

Machine vision can generate a large amount of data in individual animals with high precision and through remote sensing (non-invasive method), but there is still a need to optimize accurate data collection and individual identification/recognition (connecting images to animal ID is still a challenging process). Kashiha et al. (2013) investigated the feasibility of an automated machine vision method to identify marked pigs in a pen and achieved an accuracy of 88.7%. However, more efficient alternatives are still needed. Major and on-going advancements are happening in this area. For instance, facial recognition to identify individual animals is currently being investigated (Hansen et al., 2018).

Wurtz et al. (2019) performed a comprehensive systematic review of studies that used machine vision technology to assess behavior of indoor-housed farm animals. The authors highlighted the need to build upon existing knowledge, instead of developing devices from scratch, and validate these devices under commercial settings (in large scale). Some equipment cannot be used for measuring large numbers of animals, which is a constraint for generation of data for breeding purposes.

### Activity Sensors (Accelerometers, Activity Monitors)

Activity sensor or accelerometer devices are becoming popular in commercial livestock operations and, therefore, have great potential to generate large-scale datasets for breeding purposes. In general, accelerometers contain several sensors that record location and transmit velocity and acceleration data in one or all three dimensions (Benjamin and Yik, 2019). This includes static forces (e.g., animal is lying down), as well as movements (e.g., walking; Benjamin and Yik, 2019). These devices can be attached to different parts of the animal body (e.g., ear, neck, back, feet, and legs) and classify a variety of activities such as feeding, gait (and lameness), lying, panting, ruminating, standing, walking, nest-building (pigs), and grazing behavior (Cornou and Lundbye-Christensen, 2008; Escalante et al., 2013; Oczak et al., 2015; Thompson et al., 2016; Traulsen et al., 2018; Alsaaod et al., 2019; Benaissa et al., 2019; Halachmi et al., 2019). These metrics can be used as indicators of welfare (including health status) and for detection of positive or negative welfare status (Alsaaod et al., 2019; Benaissa et al., 2019). For instance, day-to-day variation in activity has been successfully used for lameness detection in dairy cattle (De Mol et al., 2013; Alsaaod et al., 2019), which is a heritable trait (Chapinal et al., 2013). Accelerometers and activity loggers have been also used in poultry to record the development of space use in layers housed in multi-tiers aviaries (Kozak et al., 2016a, b) and gait in grower and finisher turkeys (Dalton et al., 2016). Results reported in de Haas et al. (2017) suggest that activity patterns recorded by accelerometers can help to detect the onset of feather pecking. Therefore, recording devices such as accelerometers and activity monitors are sensitive to detect the development of behavioral and health problems in livestock.

In this section, we describe some studies using activity sensors that generated data feasible for inclusion in selective breeding schemes. The pedometer is a commonly used activity-monitoring device in dairy cattle, and there are multiple types available in the market. For instance, Shepley et al. (2017) and Mattachini et al. (2013) reported the successful application of pedometers for calculating activity and detecting lying behavior in dairy cows, respectively. Oczak et al. (2015) used accelerometer data (ear tag with a 3-axis accelerometer sensor) to determine nest-building behaviors of non-crated farrowing sows with more than 85% accuracy. This could aid in the generation of data to improve genetic selection for maternal behavior and piglet survival. Borchers et al. (2016) evaluated six different triaxial accelerometer technologies that provided accurate assessment of cow behavior, including feeding time, lying time, and rumination pattern. Along the same lines, Benaissa et al. (2019) used leg- and neck-mounted accelerometers combined with machine learning algorithms to automatically record dairy cow behavior (i.e., lying, standing, and feeding behavior) with high precision (80–99%) and sensitivity (87–99%).

Activity sensors can also be useful in outdoor production systems. For instance, González et al. (2015) performed unsupervised behavioral classification of electronic data collected at high frequency from collar-mounted motion and GPS sensors in grazing cattle. The behaviors assessed included foraging, ruminating, walking, resting, and “other active behaviors” (which included scratching against objects, head shaking, and grooming). Similar results have also been presented in other studies (e.g., Williams et al., 2016; Manning et al., 2017). As wireless data transfer in real time from collar transmitters to data analysis stations is possible and feasible, the large datasets generated are another great source of potential welfare indicators to include in pasture-based breeding schemes. In free-stall-housed dairy cattle, Bikker et al. (2014) indicated the potential use of a 3-D accelerometer that can be attached to ear identification tags and used to classify behaviors (e.g., resting, ruminating) based on ear movements.

In summary, accelerometers are small and low-cost devices that can be embedded into wearable sensors used in wireless sensor networks to generate and transfer real-time data to databases (data center stations). They are usually used for tracking animals’ positions and recording locomotion and activity/inactivity patterns in general (Benjamin and Yik, 2019), but a large number of traits can be derived from the data collected (Williams et al., 2016). In addition to using all the data generated for management (e.g., reproduction, disease detection) purposes, there is still a greater need to investigate the usefulness of such datasets for breeding more resilient animals with a better welfare. We expect that the recent availability of large-scale datasets generated by such devices in herds/flocks of animals with both pedigree and genomic information has great potential to redirect livestock breeding goals.

### Acoustic Sensing (e.g., Vocalization)

Livestock vocalizations can be a good source of information about animal welfare status and social interactions (Exadaktylos et al., 2014; Neethirajan, 2017). Acoustic sensing is a non-invasive method, inexpensive, and less dependent on lighting or the specific position of the animal (Mcloughlin et al., 2019). Some studies have investigated the relationship between vocalization and health (Exadaktylos et al., 2008; Silva et al., 2009; Ferrari et al., 2010), poultry welfare (Zimmerman and Koene, 1998), stress events [e.g., piglet crushing (Manteuffel et al., 2017), pain during husbandry procedures (Marchant-Forde et al., 2009)], and feeding behavior based on pecking sound (Aydin et al., 2014). Various devices have been developed over time. For instance, a microphone can be installed in rumination neck collars to record rumination time based on sounds of regurgitation (Ambriz-Vilchis et al., 2015).

Despite the wealth of information that can be captured by sounds, acoustic devices are a more challenging source of data collection for livestock breeding purposes. Most commercial breeding programs are of medium to large size and intensive systems (i.e., many animals are housed together at high stocking density). Various sounds are therefore produced at the same time, and sound analysis or sound recognition becomes difficult due to background noise (Du et al., 2018). Identifying the focal animal emitting the vocalization is also challenging, especially under on-farm conditions (e.g., noise background due to feeding and ventilation equipment, other animals). There are automatic measurement techniques and software being developed that could focus on specific vocalizations at specific time points (e.g., transport, handling; Moura et al., 2008; Halachmi et al., 2019). There might also be an opportunity to combine technologies such as machine vision, machine learning, and acoustic sensors.

### Automatic Milking Systems (Milking Robots)

With the intensification of dairy cattle production, automated milking systems (AMS; milking robots) are becoming more popular around the world. Labor cost savings in AMS have been estimated to range from 18 to 46% (Rotz et al., 2003; Mathijs, 2004; Bijl et al., 2007). In addition, the benefits of AMS include higher milk production per cow as a result of greater milking frequency (Tremblay et al., 2016; Tse et al., 2017), improved cow welfare (Jacobs and Siegford, 2012), earlier and easier disease detection (Tse et al., 2017), more interesting/fewer routine activities for the dairy producers (Woodford et al., 2015), and more flexible lifestyle to the farmers compared to conventional milking systems. The proportion of dairy farms using AMS is expected to increase substantially over the next years. Moreover, AMS generate a large amount of data that can be used to derive phenotypes that can be helpful for breeding purposes [e.g., disease disorders (King and DeVries, 2018)].

Several variables influence the welfare, performance, and efficiency of milk production in AMS. These traits include: (1) the willingness of the cow to voluntarily enter the milking robots. Therefore, milking interval and frequency are largely influenced by individual cow motivation. In this regard, cow training has been identified as a key challenge by producers (Tse et al., 2018). Thus, genetically selecting cows that are easier to train (or other motivation traits to enter the milking robot, such as low neophobic cows) is highly desirable; (2) cow ability to stay calm during cleaning/disinfection and attachment of milking equipment, especially in the presence of sounds and mechanical movements. Cows with a proactive temperament kick-off the milking equipment and prolong preparation and teat attachment times (Wethal and Heringstad, 2019); (3) inter-milking interval; (4) udder and individual quarter milk production (as more heterogeneous production among quarters will result in longer retention in the milking box); (5) udder conformation and teat size/placement, which is associated with teat cup attachment success rate; (6) milking time and length of the milking procedure (milking box time), which is directly associated with milking speed; (7) milk flow rate (milking speed). It is worth noting that milking speed is unfavorably correlated with udder health, and consequently, both traits need to be considered simultaneously (Sewalen et al., 2011). In addition, (8) cow dominance behavior, as more submissive cows are forced to wait for a longer period of time and forced to adjust their feeding behavior and milking times; and, (9) ability to quickly leave the milking robots after the last teat cup is removed. Despite the importance of all these traits, relatively few studies have investigated how they can be quantified based on data generated in AMS, their genomic predictive ability, and the degree to which these traits are associated with longevity, health (e.g., mastitis), and other economically and welfare important traits. This is a great source of data that can be used to genetically improve various resilience and performance traits in dairy cattle. More recently, some studies have investigated the genetic background of AMS-derived traits, indicating that it generates various variables that are heritable (Table 2).

### Individual Feed Intake Recording Systems

Individual feed intake recording systems are usually used for collecting data to enable precision management as well as genetic and genomic selection for improved feed efficiency (Hoque and Suzuki, 2009; Egger-Danner et al., 2014; Hadinia et al., 2019). However, there are additional variables that can be used as proxies of animal resilience and feeding behavior (Maselyne et al., 2015; Putz et al., 2019). For instance, voluntary variations in feed intake can indicate disease resilience, feeding competition, or negative agonistic interactions (Ahmed et al., 2014; Munsterhjelm et al., 2015; Matthews et al., 2016; Putz et al., 2019). In some cases these changes might not differ with regards to the total consumption but rather the frequency and duration of feeding activities (Tolkamp et al., 2011).

There is a large number of automated feeding systems commercially available that can be used to measure feed intake, feeding behavior, and other related variables (Hoque and Suzuki, 2009; Chen et al., 2010; Maselyne et al., 2015; Johnston et al., 2016; Matthews et al., 2016). Most systems use specially-designed single-space feeders (Maselyne et al., 2015). In general, there is a RFID (radio-frequency identification) antenna to identify the focal animals feeding and traits of their feeding bout. In addition to consumption rate (i.e., feed intake per unit of time), various other variables can be extracted such as the frequency of meals, meal duration, feeding duration, feeding pattern (e.g., time of the day; Maselyne et al., 2015), agonistic behaviors, and dominance relationships among dairy cows (Foris et al., 2019). Automatically recorded datasets have been used to understand the genomic background (including GWAS) of feeding behavior traits such as daily number of feeder visits, feeding time and duration per visit, and total daily duration at feeder (Do et al., 2013). Predictors or early indicators of tail biting outbreaks have been identified using data from electronic feeders (Wallenbeck and Keeling, 2013), suggesting another potential source of data for selective breeding against damaging and aggressive behaviors in pigs.

Automated calf feeders are becoming more common as well (Johnston et al., 2016). These systems deliver milk via a nipple at volumes and frequencies that resemble natural calf feeding behavior, support faster growth (De Paula Vieira et al., 2008), and promote calf health such as reduced sickness events (Godden et al., 2005; Barkema et al., 2015). The data generated (e.g., individual milk intake rate, frequency of feeding events) can also be used to derive proxies for genomic selection for improved calf health and overall resilience variables for genomic selection purposes.

In the case of poultry, precision feeding stations in broiler breeders can provide individual information about their performance in terms of growth rate and feed intake during rearing and lay (Hadinia et al., 2019). This information allows individual and automatic management of the feed restriction level and improve body weight uniformity in the flock (Zuidhof, 2018). For breeding selection purposes, individual performance records with precision feeding enable selection for feed efficiency. Feed efficiency in addition to other traits in the pedigree lines facilitates the selection of a parent stock with high welfare and performance that need low feed intake for the same growth rate.

In summary, automated feeding systems are becoming popular in livestock production and the large amount of data generated can also be used to derive welfare and resilience indicators for genetic and genomic selection (Howie et al., 2009, 2011). Studies investigating the genetic background of traits measured by automated feed intake recording systems are shown in Table 3.

### Microbiota Profiling

The gut microbiome can influence various host biological processes including immunity, growth, metabolism, brain development and functioning, behavioral stress (both acute and chronic), neurophysiological disorders, and emotional well-being such as anxiety and depression (Mu et al., 2016; Karsas et al., 2018; Kraimi et al., 2019). Therefore, an alternative (and complementary route) approach for minimizing welfare issues might be by altering the gut microbiota through selection (i.e., host-microbiome interactions), dietary changes (Parois et al., 2020), and management processes (Kurilshikov et al., 2017; Kraimi et al., 2019). There is evidence of a bidirectional interaction between the host and the gut microbiome in which changes in the microbial community affect host behavior and perturbations in behavior alter the composition of the gut microbiota (Collins and Bercik, 2009; Mu et al., 2016).

In pigs, the interplay between gastrointestinal tract microbiota, host genetics, and complex traits (mainly related to growth and feed efficiency) was investigated using extensive quantitative-genetic methods and they found that the bacteria genera had a significant narrow sense host heritability ranging from 0.32 to 0.57 (Camarinha-Silva et al., 2017). Another study compared the gut microbiota of two chicken lines raised under the same husbandry and dietary conditions and reported that 68 (out of 190) microbiome species were affected by genotype (line), gender and genotype by gender interactions (Zhao et al., 2013). In addition, the genetic relationships between behavior and digestive efficiency was investigated in 860 chickens from a cross between two lines divergently selected on digestive efficiency (Mignon-Grasteau et al., 2017). The authors detected common genomic regions for the presence of bacteria such as *Lactobacillus* and *L. crispatus* and traits such as feeding behavior (Mignon-Grasteau et al., 2017). A pilot study investigated the effects of early-life microbiota transplantation on feather pecking, and behavioral and physiological traits related to feather pecking (van der Eijk et al., 2020). The researchers reported that chicken lines with divergent genetic merit for feather pecking had different microbiota composition. Furthermore, early-life microbiota transplantation had immediate and long-term effects on behavioral responses and long-term effects on immune characteristics and peripheral serotonin; however, the effects were dependent on the host genotype (van der Eijk et al., 2020).

Targeted sequencing and metagenome shotgun sequencing are the two main approaches for generating microbiome profiling. Recently, a low-cost and high-throughput approach based on Restriction-Enzyme Reduced Representation Sequencing (RE-RRS) has been proposed as an alternative to capture the diversity of the rumen microbiome (Hess et al., 2020). As the costs to generate sequencing datasets decrease, microbiome profiling might be an additional relevant phenotype for further investigations and potential applications for selection to improved welfare in livestock species.

### Qualitative/Subjective Scores of Behavioral/Welfare Indicator Traits

Qualitative and subjective scoring are additional approaches to assess animal welfare. Many of these indicators can be collected on a large scale and incorporated into livestock breeding schemes to enhance animal welfare and overall resilience. For instance, Hessing et al. (1993) suggested using the back-test as a stress indicator in pigs. In brief, pigs are manually restrained on their backs for a certain period of time (e.g., 1 min) and are scored based on their behavioral responses to assess reactivity and proactivity. For example, Rohrer et al. (2013) used the back-test to determine the effects of early-life handling in pigs. In addition to the back-test, Løvendahl et al. (2005) estimated variance components for aggressive behavior of sows at mixing by counting the number of mild or severe aggressive behaviors performed or received during 30 min after grouping and determined maternal ability by recording the sows’ responses to piglet vocalization during handling.

Additional subjective scoring systems of temperament include: docility score in cattle (Adamczyk et al., 2013; Haskell et al., 2014; Schmidt et al., 2014), milking temperament in dairy cattle (Chang et al., 2020), maternal behavior and reactivity in mobile chute in Zebu cattle (Peixoto et al., 2011), and tests involving novelty, emotional reactivity, human contact and social isolation (Boissy et al., 2005; Mignon-Grasteau et al., 2017; Larsen et al., 2018). Furthermore, health scoring systems have also been proposed: lung scoring (as an indicator of pneumonia resistance, McRae et al., 2016), FAMACHA eye color chart scoring in sheep and goats [as an indicator of internal parasite resilience (Kaplan et al., 2004)], and body condition scoring (Köck et al., 2018). In addition to the objective indicators of climatic resilience presented before, some examples of qualitative scores of climatic resilience are: hair length in cattle (Piccoli et al., 2020), drooling score, respiration rate, and panting score (Gaughan et al., 2008; Schütz et al., 2014). Lameness scoring systems are widely used across livestock species (Thomsen et al., 2008; Reader et al., 2011; Nalon et al., 2013; Granquist et al., 2019). In cattle raised in extensive production systems, important adaptation traits have been genetically and genomically evaluated, including prepuce (navel) score, hair length score, and ocular pigmentation score, in addition to tick resistance (based on tick count; Piccoli et al., 2020). There are various methods available to aggregate multiple indicators to produce an overall assessment of animal welfare (Botreau et al., 2007a, b).

Despite the usefulness of qualitative scoring systems, it is important to note that observer bias and experience can influence subjective scores of animal behavior and welfare (Tuyttens et al., 2014). Fleming et al. (2016) presents a detailed description on the contributions of qualitative behavioral assessments in livestock welfare.

## Large-Scale Data Analysis: Statistical and Computational Methods

Major technological advancements in large-scale data analyses have been mainly driven by the availability and use of PLF technologies (Rutten et al., 2013). The advancements in data collection have been accompanied by the development and refinement of sophisticated statistical and data analysis methods. In this regard, a plethora of machine learning approaches have been applied (and is currently in expansion) in livestock breeding programs (Nayeri et al., 2019).

The development of prediction equations for welfare indicator traits is expected to increase. In the case of dairy species, milk MIR has a great potential to be used as indirect predictor of many traits that are expensive or difficult to measure directly, including health status indicators (De Marchi et al., 2014; Bastin et al., 2016; Dórea et al., 2018).

The wide availability of large-scale and high-throughput phenotypes requires adequate computational capacity and powerful software to store, manage, and rapidly (or real time) transfer data from farms (or other data recording stations) to central databases. High-throughput data extraction can be performed using software such as Pig^1^, MapReduce^2^, and Hadoop^3^ (Koltes et al., 2019). The definition of the methods to convert the stored phenotypes into useful information for real-time management decisions in the farm or breeding purposes is still a challenging task (Koltes et al., 2019). Therefore, the development of statistical methods such as machine learning and neural artificial intelligence are of great relevance.

Phenotypic quality control is one of the first steps in the data analysis process and consists of removing noise and outliers. Data standardization or transformation can also be needed depending on the statistical model assumptions, when merging datasets from different populations, or when using different equipment, calibration methods, or data collection protocols (Norton and Berckmans, 2018). Big data handling and manipulation requires good computational infrastructure and efficient programming methods (Nayeri et al., 2019). Furthermore, most PLF devices generate repeated records for each individual [i.e., longitudinal traits (Oliveira et al., 2019a)], which are highly desirable for monitoring livestock welfare. However, the covariance structure among records needs to be considered in the statistical models (Oliveira et al., 2019a).

Defining the appropriate statistical methods and models to be used for data analyses is paramount for the accuracy of the results obtained. However, this can be challenging when there is a large number of variables extracted from the high-throughput phenotypic datasets (Koltes et al., 2019; Nayeri et al., 2019). In the case of predictive modeling, feature selection can improve model performance and avoid or reduce model overfitting (Saeys et al., 2007), as well as improving the model interpretability (Butterworth, 2018).

One approach to analyze high-throughput phenotypic data consists of statistically evaluating differences between the averages of groups (Norton and Berckmans, 2018), considering all together or within specific time points. Thus, the research question needs to be clearly described, which is directly related to the final goal of using the monitoring algorithm (Nayeri et al., 2019). Common examples of welfare-related objectives are recognizing cow gait score or footpad lesion scores in chickens (Norton and Berckmans, 2018). The next step consists of defining the reference points that can be used to draw a conclusion related to the final algorithm-use goal (Butterworth, 2018).

When fitting longitudinal records, many popular statistical methods will frequently overfit the data, due to its high dimensionality and rank deficiency (Butterworth, 2018). In this context, machine learning is viewed as a key method to deal with big data, and it has proven to be useful in classifying individuals through supervised learning algorithms (Nayeri et al., 2019). The classification methods based on supervised learning algorithms can use class labels previously defined by the researcher, or by permitting the unsupervised learning (Saeys et al., 2007). However, other methods such as neural networks, support vector machines, linear and non-linear density based classifiers, decision trees, naive Bayes, wavelet analysis, k-nearest neighbor, and k-means have also being reported in the literature in terms of classification analysis (Butterworth, 2018; Koltes et al., 2019; Nayeri et al., 2019). For instance, Bakoev et al. (2020), evaluated the prediction accuracy of nine machine learning classification algorithms and reported that Random Forest and K-Nearest Neighbors better predicted pig leg weakness based on measurements taken at an early stage of the animal development.

## Genetic and Genomic Selection to Enhance Animal Welfare and Overall Resilience

There are two main options to evaluate animal welfare (based on resilience indicators) in a breeding program (Knap, 2008): (1) using reaction norm analysis, which enable the estimation of breeding values for production performance considering different environmental gradients (indirect approach), or, (2) directly including the measurable welfare traits in the breeding goal and in the selection indexes (direct approach), as mentioned in the previous sections in this review. However, usually reaction norms have been used for genetic evaluations of livestock animals due to the arduousness of using the direct approach and correctly defining the measurable trait (Rauw and Gomez-Raya, 2015).

Reaction norm has been defined as the expression pattern of a trait along a continuous environmental gradient (de Jong, 1995; Knap, 2005). Several variables can be used as environmental gradients in the reaction norms, such as disease exposure, social stress, temperature, and nutrient quality (Rauw and Gomez-Raya, 2015). Thus, animals maintaining production, health, and coping well across the environmental gradient are suggested to be more resilient (Rauw and Gomez-Raya, 2015). Although reaction norms are mostly described as linear relationships, they can take more complex shapes. Thus, the first derivative of the function in that environment is defined as plasticity, i.e., the difference in trait measurements between environments (de Jong, 1995).

Reaction norm models have been mainly applied to beef and dairy cattle, due to the wide use of artificial insemination and consequently dispersion of semen into several different environments. Therefore, this wide range of environments facilitate the investigation of changes in the expression of traits through a continuous descriptor of environments (Rauw and Gomez-Raya, 2015). In this context, Ravagnolo and Misztal (2002) estimated the genetic component of heat tolerance for non-return rate in Holstein cattle using a random regression animal model (Oliveira et al., 2019a) and temperature humidity index (THI). THI was calculated using temperature and humidity data provided by public weather stations, which can be obtained from on-line sources in various countries. For instance, this has been done in beef cattle for birth weight, weaning weight, post-weaning weight gain, and yearling scrotal circumference by using reaction norms and the contemporary groups as the environmental descriptor (Santana et al., 2013).

Another interesting application of reaction norms is for genomic prediction of breeding values. Few studies have reported the estimation of breeding values for animals in different environments using either a multiple-step (Silva et al., 2014) or single-step (Mota et al., 2016; Oliveira et al., 2018) approach. In this context, Silva et al. (2014) concluded that reaction norms should be used for proper genomic evaluation of total number of piglets born. Moreover, Oliveira et al. (2019b) showed that random regression models can be used to estimate Single Nucleotide Polymorphism (SNP) effects over time in genome-wide association studies.

Despite the great potential of reaction norm models for genetic and genomic evaluation of livestock animals, they have not been used to model welfare indicators yet. However, Sih et al. (2004) proposed that behavior can be included in reaction norms models. Similarly, Dingemanse et al. (2010) indicated that animal behavior can be described as a function of environmental variation. In this context, Dingemanse et al. (2012) used reaction norms to estimate genetic parameters for exploration behavior in an open-field test of wild-caught three-spined stickleback fish. Similar analysis can potentially be applied to social interactions, feeding behavior, and activity patterns in livestock production systems (Rauw and Gomez-Raya, 2015). In addition to using climatic variables from public weather stations, there is a growing interest on recording additional and more precise climatic variables within production operations (Laberge and Rousseau, 2017).

As reviewed by Egger-Danner et al. (2014), some countries have well established health recording systems (e.g., Austria, Canada, France, Germany, and Nordic countries), including the use of veterinary diagnoses, whereas others focus on producer-recorded data. Combined use of health data from farmers and diagnosis documented by veterinarians may be an option to improve coverage of direct health data (Egger-Danner et al., 2014). Data recorded in slaughter facilities (e.g., tail lesions in pigs; skin lesions in poultry) might also be a useful source of data for breeding purposes.

Modifying animals’ environments by eliminating all stressors and other causes of poor welfare through management approaches (e.g., housing, management practices, nutrition, biosecurity) can be thought of as the soundest alternative to improve welfare in livestock operations. However, this is very difficult (or impossible) to achieve in commercial farms due to economic and practical constraints and additional factors such as climate change and antibiotic resistance. Therefore, genetically selecting animals that are more resilient to different stressors and better suited for that environment, while also developing strategies to minimize the stress sources and causes of impaired welfare, is likely to be the more successful alternative in the long-term (Rodenburg and Turner, 2012).

There is clear within-population genetic variation to response to stress and overall resilience (Tables 1–4), indicating that genetic progress for enhanced animal welfare can be successfully achieved. Direct selection for reduced stress responsiveness can impact other relevant traits (e.g., performance, reproduction) due to pleiotropic or linkage effects. Therefore, the practical application of selective breeding to enhance welfare and overall resilience will require the use of selection indexes to enable simultaneous genetic progress on all relevant traits in individual populations. Ignoring genetic correlations among traits can result in undesirable effects, such as reduced welfare, coping mechanisms, and overall resilience due to primary selection for performance traits (Rauw et al., 1998; Rauw and Gomez-Raya, 2015). Furthermore, ignoring direct selection for welfare indicators could increase competition and agonistic interactions, which would reduce welfare, and consequently, overall productivity (Cheng, 2010; Rodenburg and Turner, 2012; Muir et al., 2014).

Genetic and genomic selection to enhance animal welfare and overall resilience can be achieved through multi-trait selection and selection indexes (Muir et al., 2014), combining various indicators of welfare and resilience, as described in this review. These traits include both direct and indirect indicators of welfare and resilience. Genomic selection has become the gold standard approach for genetically evaluating and selecting breeding animals (Meuwissen et al., 2016). This is especially advantageous for welfare traits because genomic breeding values can be predicted for selection candidates that have not been challenged by a certain stressor (e.g., pathogens, heat stress). This can be done by using data from a large training population (animals with both phenotypes and genotypes) of individuals genetically related that are raised under those stress conditions (e.g., tropical regions in the case of heat stress). Genomics also provides an opportunity to better understand the biological mechanisms associated with each trait through genome-wide association studies and functional analyses. In addition to genomic and phenotypic datasets, alternative “-omic” approaches can be of great value to unravel biological mechanisms underlying animal welfare and to improve the accuracy of genomic predictions. This includes multiple phenotypic layers, such as gene expression (transcriptomics), epigenomics (e.g., DNA methylation), proteins (proteomics), metabolites (metabolomics), lipids (lipidomics), and microbiota (microbiomics). The integration of multi-omic data and joint modeling and analyses are very powerful techniques to understand the systems biology of healthy and sustainable production of animals (Suravajhala et al., 2016). Despite the usefulness of such approaches, there are still many challenges and further developments to be addressed (Suravajhala et al., 2016).

Welfare is predicted to play an important role in livestock breeding goals (Rodenburg and Turner, 2012; Croney et al., 2018a). This is mainly due to the clear benefits of improved welfare in farm production efficiency and sustainability [e.g., reduced mortality, improved animal health, and product quality (Dawkins, 2017)], but in certain cases can have detrimental effects in overall production efficiency. In this context, various livestock breeding programs have started to incorporate welfare and resilience indicators in their breeding programs. Examples of welfare indicators that have been investigated or included in selection schemes in livestock breeding programs around the world are: aggression (Løvendahl et al., 2005); behavior (Rohrer et al., 2013); boar taint (to avoid castration; Tajet et al., 2006; Zadinová et al., 2016), calf wellness (Gonzalez-Peña et al., 2019), calving ease (Jamrozik and Miller, 2014; Vanderick et al., 2014; Li and Brown, 2016), cortisol levels (Mormède et al., 2011); docility (Norris et al., 2014); feather pecking (Dawkins and Layton, 2012), feet and leg health (Kapell et al., 2012, 2017); fertility disorders (Guarini et al., 2018; Fleming et al., 2019), hoof health [in cattle (Chapinal et al., 2013; Häggman and Juga, 2013; Heringstad et al., 2018), sheep (Conington et al., 2008), and pigs (Quintanilla et al., 2006)]; lesion scores (Wurtz et al., 2017; Angarita et al., 2019), longevity (Serenius and Stalder, 2006; Ramos et al., 2020), mastitis (Martin et al., 2018); maternal behavior and progeny survival (Gäde et al., 2008; Hellbrügge et al., 2008a, b), metabolic diseases (Egger-Danner et al., 2014; Jamrozik et al., 2016; Pryce et al., 2016), nematode resistance (Doeschl-Wilson et al., 2008), overall resilience (Berghof et al., 2019), paratuberculosis (Brito et al., 2018; Mallikarjunappa et al., 2020); pre-weaning survival (Su et al., 2007; Nielsen et al., 2013); social dominance (Tong et al., 2020); tail or ear biting (Breuer et al., 2005), and thermal tolerance (Fragomeni et al., 2016; Misztal, 2017; Nguyen et al., 2017; Xu et al., 2017). Genetic selection and modern genomic techniques (e.g., gene editing) might also be an alternative to eliminate the need for stressful procedures in commercial applications such as cattle dehorning (Van Eenennaam and Young, 2018).

Since domestication, artificial selection has altered coping mechanisms of livestock animals. For instance, there is evidence that chronic stressors have made modern laying hens more fearful of humans than their ancestors (Jones et al., 1988; El-Lethey et al., 2000; Jensen et al., 2006), and increased feather pecking and cannibalism in a larger range of environmental conditions (Canario et al., 2013; Decina et al., 2019). Also, pigs selected for high lean growth, show increased anxiety in the presence of humans (Scott et al., 2000) and leaner pigs are more stressed by transport and harder to handle than fatter pigs (Grandin, 1998). In general, livestock breeding programs focus primarily on direct breeding values (selection for individual production; Rodenburg and Turner, 2012). However, most livestock species are group-housed, and therefore, genetic selection for associative effects (social breeding values) has been proposed (Muir, 2003). Associative effects represent the social impacts of one animal on the performance of another. For instance, genetic selection based on group rather than individual performance can reduce mortality due to aggressive behaviors in poultry and pigs (Muir, 1996, 2005; Rodenburg et al., 2010; Angarita et al., 2019). The incorporation of indirect genetic effects in livestock breeding programs has the potential to substantially increase responses to selection in traits affected by social interactions [e.g., feather pecking, cannibalism; (Rodenburg et al., 2010; Rodenburg and Turner, 2012)]. There are three main methods to improve associative effects (Ellen et al., 2014): (1) direct selection to reduce aggressiveness; (2) multi-level selection (Bijma and Wade, 2008; Muir et al., 2013); and (3) multi-trait selection where the direct and associative effects of each animal are estimated and directly selected for in a selection index (Muir, 2005; Bijma et al., 2007a, b; Muir et al., 2014). Some factors that can impact the estimates of indirect genetic effects are: level of competition for resources (e.g., feed, water), stocking density, age, and body weight variation when animals are mixed.

As previously indicated, selective breeding for enhanced welfare may require breeding animals to be exposed to the stressor on which the animals will be genetically evaluated for (e.g., pathogens, thermal stress). However, breeding nucleus animals are usually raised under high health and biosecurity standards, in low stocking densities, and low level of environmental stressors. Therefore, there might be genotype-by-environment (GxE) interactions if selection is based entirely on phenotypic records obtained in nucleus farms. Genomics can facilitate this process, as a training population can be developed based on animals raised in commercial farms (with all common stressors). Therefore, GxE should be considered when performing genomic selection for improved animal welfare and overall resilience. For example, behavior expression might differ based on animal group size (even at the same stocking density), resource availability, housing system, and use of PLF technologies (e.g., milking robot).

Practical implementation of selection to enhance animal welfare will require the development of appropriate selection indexes for combining indicators of welfare and overall resilience. However, this is challenging due to the difficulty of determining the economic value or importance of each welfare indicator trait (Nielsen et al., 2008; Croney et al., 2018a). In this context, the main challenges associated with the incorporation of animal welfare in livestock breeding goals are (Nielsen et al., 2008): (1) defining the social and economic value of improved animal welfare; (2) the perspectives of all stakeholders (e.g., farmers, consumers, citizens, and governmental authorities) need to be considered when defining the breeding goals, in which a consensus can be difficult to be achieved; and, (3) potential antagonist relationships with performance (or other conventional traits; Nielsen et al., 2008).

The wealth of data generated by PLF, data recording organizations, and genotyping schemes require the availability of good computational infrastructure, efficient software and well-trained professionals (Morota et al., 2018; Koltes et al., 2019). In addition to management practices, using these datasets for breeding purposes is expected to motivate farmers to further invest in phenotyping and genotyping tools. More efficient use of PLF datasets include international modeling and data-sharing initiatives and by adopting a collaboration model between industry, researchers, farmers, and stakeholders (Halachmi et al., 2019).

Most studies and applications of breeding for animal welfare have focused on intensive production systems, whilst extensive conditions (infrequent handling or reduced contact with humans) have largely been ignored (Turner and Dwyer, 2007; Rodenburg and Turner, 2012; Turner et al., 2018). There are welfare issues in extensive production systems (e.g., heat stress; temperament; and disease challenge), and genetic selection for improved welfare under those conditions should also be *a priority* for breeding companies and organizations.

Agroecological and organic production systems are expected to become more common over the next decades (Dumont et al., 2014; Phocas et al., 2016a, b). Therefore, breeding goals will also need to be refined for improved welfare and resilience under those conditions (as reviewed by Phocas et al., 2016a, b). As noted by Phocas et al. (2016a), breeding objectives for smallholder production systems in developing countries tend to differ from those in developed countries, especially due to environmental, economic and socio-cultural differences. Therefore, it is clear that welfare concerns are present across production systems, but in different levels, and alternative approaches will need to be taken to optimize welfare while increasing food production to meet the demands of a growing human population.

## Final Remarks

Quantifying welfare is paramount for breeding more resilient animals. Some of the main requirements for defining ideal welfare indicators are: (1) variables should be continuously recorded throughout the animals’ life; (2) a large number of variables need to be accurately measured in individual animals as biological indicators of the five freedoms, including physiological, behavioral, and emotional state, and physical and health characteristics; (3) data collection should be based on non-invasive methods that do not result in additional stress or discomfort to the animals or alter their routine or circadian rhythms; (4) the derived phenotypes need to be collected at a low cost to enable measurement of a large number of animals, which is a requirement for successful implementation of genetic and genomic evaluations; (5) phenotypic measurements that are accurate, valid, repeatable, and comparable among laboratories, countries, or companies is critical; and (6) the phenotypes identified need to be heritable and repeatable.

The definition of welfare indicators is largely dependent on a clear understanding of the biological and emotional mechanisms behind the phenotypic variability observed in the animal’s response to different stimuli. Therefore, the evaluation of animal welfare involves a complete assessment of the animal’s physiological, behavioral, physical, and emotional state. Some of these indicators can even be quantified prior to clinical signs of poor welfare (e.g., clinical mastitis).

The rapid development of integrated biological (e.g., *-omics* technologies) and engineering systems and the IoT is enabling the development of affordable monitoring devices and high-throughput technologies (Neethirajan et al., 2017). These tools can be used to individually monitor large numbers of animals in commercial settings and are advantageous to quantify biological indicators through rapid, repeatable, and automated measurements. The technological devices used include sensors such as cameras, microphones to capture vocalizations, thermometers, automated feeding and milking systems, automatic scales to measure lean-fat ratios, milk spectral data, electrodes to detect skin conductivity and heart rate, and accelerometers. Qualitative scoring systems can also be used to assess some aspects of animal welfare as well as data routinely collected in commercial farms. As Animal Welfare science evolves, novel indicators will emerge and improve our understanding of animal welfare. Further improvements in precision technologies, integration of data from multiple systems and, in particular, increased training of farmers, their personnel, and advisors to use sensor derived data will play a major role in modern livestock production (Barkema et al., 2015). The greater availability of high-throughput phenotyping technologies (e.g., automated monitoring systems) in nucleus and commercial farms, better communication and data sharing among data recording organizations (e.g., Dairy Herd Improvement, breed associations, veterinary clinics, and slaughter facilities), and greater integration of complementary disciplines will contribute to overcoming some of the challenges associated with time and cost of welfare data collection (Wemelsfelder and Mullan, 2014). In addition, PLF tools enable the collection of continuous and real-time phenotypes as well as environmental conditions (e.g., thermal stress, humidity, and air quality; Laberge and Rousseau, 2017), that are of great use for assessing animal welfare.

Genetic and genomic selection to enhance animal welfare and overall resilience can be achieved through multi-trait selection and selection indexes (Muir et al., 2014), combining various indicators of welfare and resilience. Genomic selection is especially advantageous for welfare traits because genomic breeding values can be predicted for selection candidates that have not been challenged by a certain stressor (e.g., pathogens, heat stress). Genomic selection for welfare traits, itself, is unlikely to solve all the welfare issues in commercial livestock operations. However, selective breeding is a complementary approach to other strategies (e.g., management, nutrition, housing, and biosecurity), which will result in permanent and cumulative gains in welfare (resilience) over generations.

Genetic and genomic selection for improved animal welfare require a multidisciplinary approach, including the integration of a multitude of scientific field such as cell and molecular biology, neuroscience, immunology, stress physiology, computer science, engineering, quantitative genomics, and bioinformatics. In this context, it is paramount to train the next generation of researchers in multi-disciplinary teams and develop collaborative research projects.

High welfare standards will continue to be *a priority* in livestock production systems. We expect that this review provides a comprehensive description of welfare phenotyping techniques coupled with the use of genetic and genomic selection to enhance animal welfare in commercial production systems.

## Author Contributions

LB conceived the article subject, prepared the review outline, and developed the concepts during the writing process. LB, HO, BM, JJ, and AS wrote the manuscript. LB, HO, BM, JJ, AS, AA, and JM-F edited the manuscript and provided additional comments. All the authors revised and accepted the final version of the manuscript.

## Conflict of Interest

The authors declare that the research was conducted in the absence of any commercial or financial relationships that could be construed as a potential conflict of interest.

## Footnotes

1
https://pig.apache.org/

2
https://en.wikipedia.org/wiki/MapReduce

3
https://hadoop.apache.org/
